# Deciphering the mechanoresponsive role of β-catenin in keratoconus epithelium

**DOI:** 10.1038/s41598-020-77138-3

**Published:** 2020-12-07

**Authors:** Chatterjee Amit, Prema Padmanabhan, Janakiraman Narayanan

**Affiliations:** 1 Department of Nanobiotechnology, KNBIRVO Block, Vision Research Foundation, Sankara Nethralaya, 18/41, College Road Nungambakkam, Chennai, Tamil Nadu India; 2 School of Chemical and Biotechnology, SASTRA, Deemed University, Tanjore, Tamil Nadu India; 3 Department of Cornea, Medical Research Foundation, Sankara Nethralaya, Chennai, Tamil Nadu India

**Keywords:** Biomarkers, Physiology

## Abstract

Keratoconus (KC) is a corneal dystrophy characterized by progressive ectasia that leads to severe visual impairment and remains one of the leading indications for corneal transplantation. The etiology is believed to be multifactorial and alterations have been documented in the biomechanical, biochemical and ultrastructural characteristics of the cornea. While the exact site of disease origin is still debated, changes in the corneal epithelium are believed to occur even before the disease is clinically manifested. In this study we investigate the possible role of β-catenin as mechanotransducer in KC corneal epithelium. The sheets of corneal epithelium removed from keratoconic eyes when they underwent collagen crosslinking as a therapeutic procedure were used for this study. The healthy corneal epithelium of patients undergoing Laser Refractive Surgery for the correction of their refractive error, served as controls. Immunoblotting and tissue immunofluorescence studies were performed on KC epithelium to analyse the expression and localization of β-catenin, E-cadherin, ZO1, α-catenin, Cyclin D1, α-actinin, RhoA, and Rac123. Co-immunoprecipitation of β-catenin followed by mass spectrometry of KC epithelium was performed to identify its interacting partners. This was further validated by using epithelial tissues grown on scaffolds of different stiffness. Histology data reported breaks in the Bowman’s layer in KC patients. We hypothesize that these breaks expose the epithelium to the keratoconic corneal stroma, which, is known to have a decreased elastic modulus and that β-catenin acts as a mechanotransducer that induces structural changes such as loss of polarity (Syntaxin3) and barrier function (ZO1) through membrane delocalization. The results of our study strongly suggest that β-catenin could be a putative mechanotransducer in KC epithelium, thus supporting our hypothesis.

## Introduction

The mechanical properties of biological tissues are essential for their physiological functions^1^ and their regulation plays an important role in driving various pathological consequences^2^. Such regulation is based on mechanotransductional processes which initiate distinct signalling pathways^1^. The cell specific and molecular basis of mechanosensing and the sequence of biochemical reactions that ensue, have been the focus of recent research^3^. Several molecules have been proposed as possible mechanosensors such as integrins, ion channels, G-protein-coupled receptors, and cell–cell junctional proteins^4^.

Keratoconus (KC) is a bilateral progressive visually debilitating clinical condition characterized by abnormal thinning and protrusion of the cornea in response to biomechanical instability of the corneal stroma. Several studies have shown that genetic, biochemical and biomechanical factors are involved in the pathogenesis of this disease^5,6^. RNA sequencing studies on KC corneal epithelium have reported collagen degradation, and downregulation of TGF β, Hippo, Hedgehog, Notch1 and Wnt signalling pathways^7,8^. The potential role of long non coding RNA has also been explored in KC^9^. While the exact site of origin of the disease is still debated, changes in corneal epithelium, basement membrane, Bowman’s layer and stroma have been reported^10,11^. Tear proteome analysis by electrophoretic separation and Mass spectrometric (MS) techniques have identified decreased expression of cystatin family of proteins (natural inhibitor of cysteine proteinases) and increased expression of lipocalin family of proteins (tear lipid binding proteins), Cathepsin B and higher level of cytokines^12^. Recent research has also highlighted the influence of endocrine function in regulating metabolism and inflammatory pathways in KC suggesting KC to be a partially systemic disease^13^. Metabolites including hormones such as prolactin-induced protein have been proposed to be important hormonally regulated biomarkers in KC^14^. While the exact etiology of the disease remains an enigma, the final common pathway is believed to be a decrease in the elastic modulus of the stroma. The lower stiffness of stroma initiates a cascade of events that results in morphological and biochemical changes that affect the shape and the optical function of the cornea. Morphological changes in the corneal epithelium have been well documented and are considered to be one of the earliest detectable changes observed in KC^15^. Based on the hypothesis that these changes are representative of a mechanotransduction process, we believe that studying the signalling pathway in the epithelium of KC patients would offer a unique opportunity to unveil the molecular basis of mechanotransduction in corneal epithelium.

Wnt/β-catenin pathway, which is known to control diverse cell functions^16^, responds significantly to extracellular matrix stiffness^17^. The central molecule in Wnt/β-catenin pathway is β-catenin which is an evolutionarily conserved protein and exists in three different subcellular locations: membrane bound, cytosolic, and nuclear^18^. The membrane bound β-catenin has adhesion function whereas its nuclear form plays an important role in transcriptional regulation. In the membrane, β-catenin binds with both E-cadherin and α-catenin which in turn interact with actin^19^. The α-/β-catenin interaction is regulated by small GTPases—RhoA, Rac1 and Cdc42, which in turn regulate actin polymerization^20^. In corneal limbal epithelial stem cells, the nuclear localization of β-catenin is reported to mediate cell proliferation. In contrast, the central corneal epithelium exhibits membrane localization of β-catenin indicating reduced cellular proliferation^21^.

Wnt signalling has been reported to be altered in KC epithelium^7^. However, to the best of our knowledge, the role of β-catenin in KC has not been explored so far. We hypothesize that β-catenin plays an important role as a mechanotransducer and that the detection of its localization could potentially be used for early diagnosis of KC. In this study, we analysed the localization and expression of β-catenin in KC epithelium. We also studied the expression of its interacting proteins such as E-cadherin, α-catenin, α- actinin, ZO1(Zonula occludens1). Scanning electron microscopy (SEM) and Actin staining revealed the structural changes in KC epithelium. β-catenin Co-IP in corneal epithelium of mild KC and healthy control identified novel interacting partners. It also explained the possible role of β-catenin in KC epithelium where canonical Wnt pathway is known to be inactive. Further, the response of β-catenin to different elastic modulus was studied using Poly dimethysiloxane (PDMS) and gelatin hydrogel with control corneal epithelium. The results further validated our hypothesis that β-catenin does have a role in mechanotransduction.

## Results

### β-catenin expression and its cellular localization in KC epithelium

**Figure 1:**
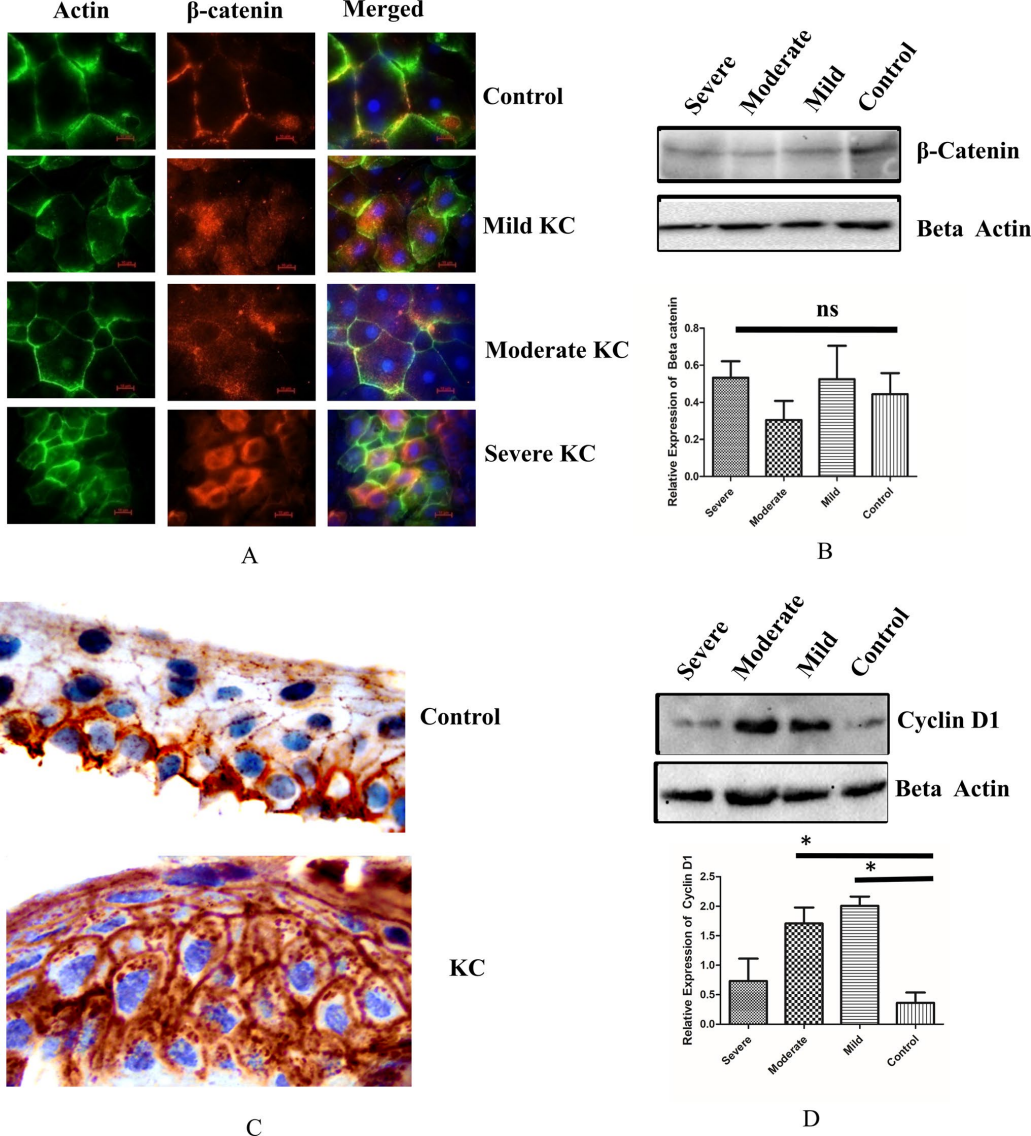
**(A)** Tissue immunofluorescence of β-catenin showing changes in localization in control, mild, moderate and severe keratoconic epithelium (n = 7) Scale bar − 10 µM. **(B)** Immunoblotting of β-catenin in control and KC tissues (mild, moderate and severe) (Full blot was cut in two halves for probing βcatenin and beta actin epi white merged image is represented in Fig S6). **(C) **Immunohistochemistry of β-catenin in control and KC tissues (n = 5). (**D**) Immunoblotting of cyclin D1 in control, and KC tissues (mild, moderate and severe) (Full blot was probed with cyclin D1 and then with beta actin, epiwhite merged image is represented in Fig S7). One way ANOVA Asterisks *, **, and *** denote a significance with *p* Values < 0.05, 0.01, and 0.001 respectively**.**

KC is a condition where a progressive degradation of collagen fibrils occurs and decreases the stiffness of the stroma. Hence, we investigated the localization of β-catenin throughout the spectrum of disease severity. The clinical details of the samples are presented in Table 1. In control epithelium, β-catenin showed membrane localization whereas in mild, moderate and severe KC, β-catenin was membrane delocalized and found to be present in both the cytosol and possibly in the nucleus as well (Fig. 1A). We analysed the expression of total β-catenin protein (*P* > 0.05) across KC epithelium and compared it with control epithelium, we did not observe any significant changes in total β-catenin expression in KC epithelium across the grades (Fig. 1B). Further to confirm immunofluorescence data, we performed immunohistochemistry. The results indicated that in control epithelium, β-catenin was membrane bound in the basal cells whereas in KC epithelium it was membrane delocalised to cytosol as well as possibly to nucleus in both basal cells and superficial cells (Fig. 1C). YAP is a known interacting partner of β-catenin and has been shown to be an important regulator of mechanotransduction of corneal epithelium^22,23^. We also analysed YAP localization using immunohistochemistry. Our data (Fig S1) showed that YAP was localized in both basal cells and superficial cells of control epithelium, where as in KC epithelium, YAP expression was found to be reduced in both basal cells and superficial cells which needs further investigation.

**Table 1:** Patient details age, keratometry and pachymetry, Km- Mean keratometry reading in Diopters.

Clinical details	Control	Mild	Moderate	Severe
Age	19.5 ± 2.9	22.85 ± 1.89	24.33 ± 2.56	25.54 ± 3.56
K1(Diopters)	44.26 ± 0.653	45.18 ± 0.423	49.81 ± 0.765	50.21 ± 1658
K2(Diopters)	42.451 ± 0.895	47.39 ± 0.722	50.46 ± 0.989	54.68 ± 0.989
Km(Diopters)	43.355 ± 0.723	46.285 ± 0.651	50.135 ± 0.835	52.446 ± 0.135
Corneal Thickness (µM)	512 ± 21.49	437 ± 31.49	410 ± 30.78	375 ± 29.05

Hence, we conclude that the localization changes of β-catenin was independent of its total protein expression. Further to validate the observation of immunofluorescence we studied the expression of β-catenin target gene cyclin D1 that has a role in cellular proliferation. In mild KC epithelium a significant upregulation of cyclin D1 (*P* < 0.05) was observed compared to control epithelium (Fig. 1D). This confirms that in KC epithelium delocalization of β-catenin from membrane to cytosol and possibly to nucleus increases the cyclin D1 expression.

### Cadherin-Catenin complex in KC epithelium

**Figure 2:**
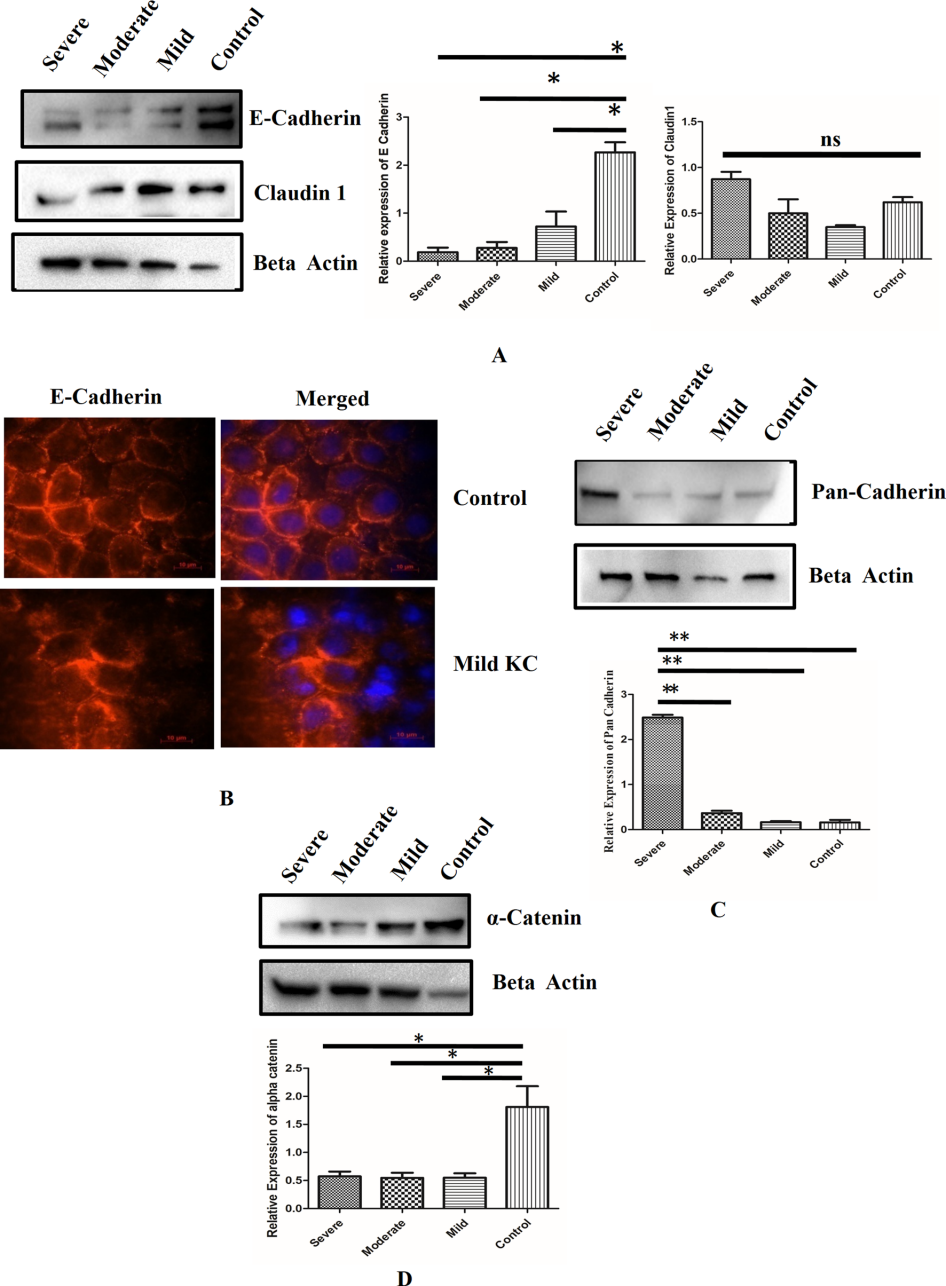
**(A)** Immunoblotting of control, mild, moderate and severe keratoconic epithelium with E cadherin and Claudin1 (n = 7) **(**Full blot was cut in three halves for probing E cadherin, claudin and beta actin image is represented in Fig S8) **(B)** Tissue immunofluorescence of E cadherin in control and mild keratoconic epithelium (n = 7) Scale bar-10 µM **(C)** Immunoblotting of Pan cadherin in control, mild, moderate and severe keratoconic epithelium.(n = 7).(Full blot was cut in two halves for probing pan cadherin and beta actin image is represented in Fig S9). (D) Immunoblotting of α-catenin incontrol, mild moderate and severe keratoconic epithelium.(n = 7)(Full blot was cut in two halves for probing alpha catenin and beta actin image is represented in Fig S10).One way ANOVA Asterisks *, **, and *** denote a significance with *p* Values < 0.05, 0.01, and 0.001 respectively.

We observed the loss of membrane bound β-catenin in KC epithelium (Fig. 1A), Hence, we investigated the expression of E cadherin, which is a binding partner of β-catenin. A significant down regulation of E cadherin in mild, moderate and severe KC epithelium (*P* < 0.05), was observed compared to control epithelium(Fig. 2A). However loss of total E cadherin did not induce any significant changes in localization of E cadherin. (Fig. 2B). Loss of E cadherin in KC epithelium prompted us to investigate tight junction protein Claudin1. However, we did not observe significant changes of Claudin 1 in KC epithelium (*P* > 0.05) (Fig. 2A). The loss of E cadherin was probably circumvented by significant upregulation of Pan cadherin levels in KC epithelium compared to control (*P* < 0.01) (Fig. 2C). Further, we analysed the expression of α-catenin which is an obligate component of epithelial adherence junctions. we observed significant downregulation in mild, moderate and severe KC epithelium compared to control (*P* < 0.05) (Fig. 2D). Possibly due to change in β-catenin localization, α-catenin expression was found to be significantly downregulated in KC epithelium.

### Morphology and cytoskeleton arrangement of Keratoconic corneal epithelial tissues

**Figure 3:**
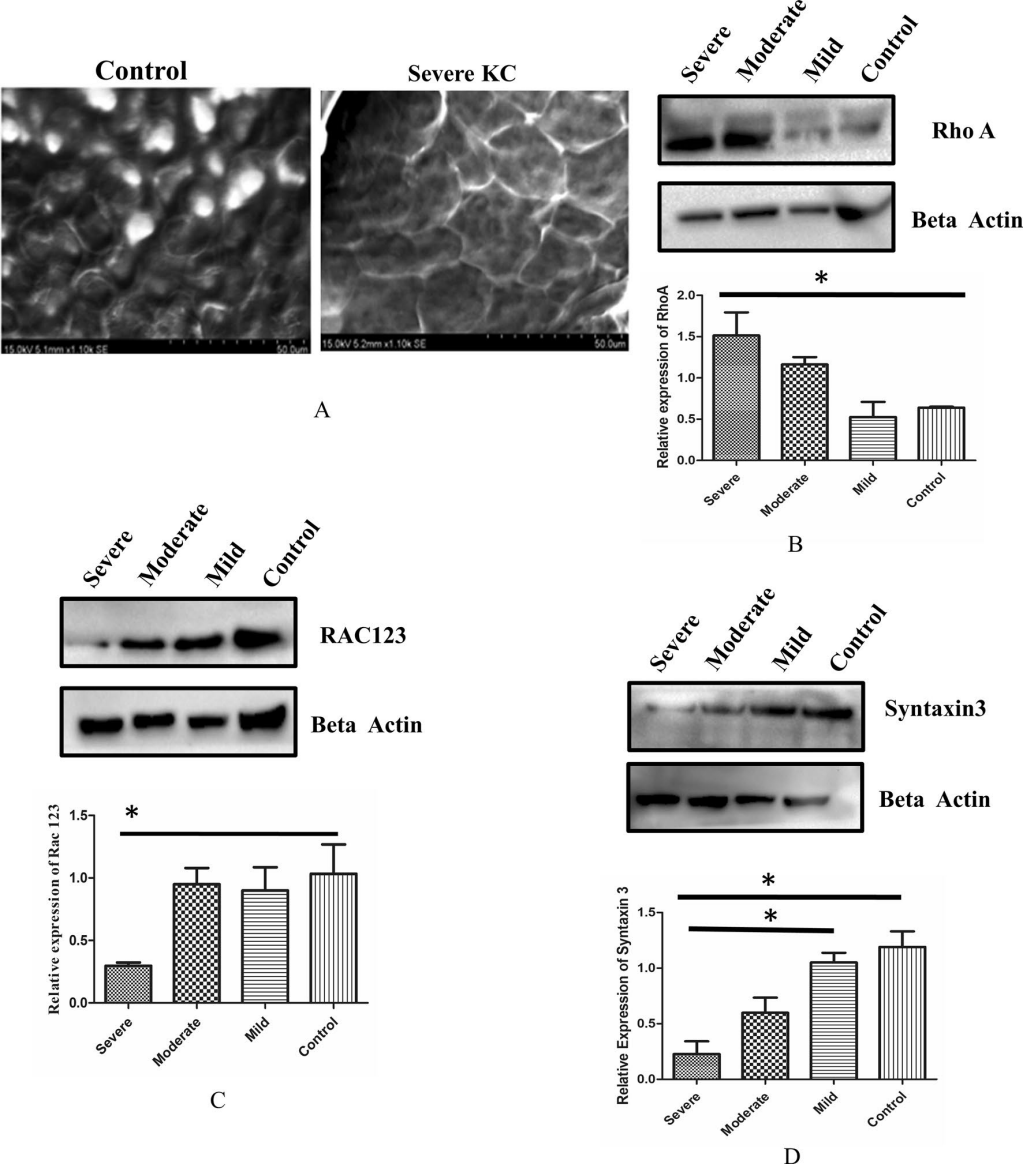
**(A)** Scanning electron microscopy of control epithelium and severe keratoconic epithelium (n = 7) Scale bar-50 µM **(B)**Immunoblotting of control, mild, moderate and severe keratoconic, RhoA (n = 7) (Full blot was cut in two halves for probing RhoA and beta actin image is represented in Fig S11) **(C)**Immunoblotting of control, mild moderate and severe keratoconic epithelium with Rac123 (n = 7) (Full blot was cut in two halves for probing Rac123 and beta actin image is represented in Fig S12) **(D)** Immunoblotting of control mild moderate and severe keratoconic epithelium with STX3 (n = 7) (Full blot was cut in two halves for probing Syntaxin 3 and beta actin image is represented in Fig S13). One way ANOVA, Asterisks *, **, and *** denote a significance with *p* Values < 0.05, 0.01, and 0.001 respectively.

The loss of epithelial integrity is known to induce structural changes in the epithelium. Therefore, we assessed the morphological and cytoskeletal changes using SEM and actin staining. Control epithelium showed uniform thickness (6 to 7 layers) in SEM imaging whereas the severe KC epithelial showed a thin layer. The basal cells of severe KC epithelium exhibited larger surface than the control epithelium (Fig. 3A). The border of KC epithelium was thinner than control epithelium. Cellular morphology and the size of the cells were altered in severe keratoconus compared to the control epithelium (Fig. 3A). Grade wise (Mild, Moderate and Severe) analysis of stress fibers in KC epithelium indicated the presence of different types of stress fibers. In mild KC epithelium, stress fibers were located mostly at the membrane of the cells whereas in severe KC epithelium the stress fibers were found across the cells suggesting the changes in the actin polymerization (Fig S2). The altered actin polymerization is known to be regulated by Rho family of proteins. Hence, we studied the expression of RhoA, Rac 123 expression in KC epithelium. Expression of RhoA was found to be significantly upregulated (*P* < 0.05) in severe KC epithelium compared to the control whereas Rac 123 levels were significantly downregulated (*P* < 0.05)in severe KC epithelium (Fig. 3B,C). The altered morphology and expression of actin polymerizing regulating proteins prompted us to analyse the polarity of KC epithelium. STX3 (Syntaxin 3) localizes to apical plasma membrane and is also involved in membrane fusion of apical trafficking pathway. The expression of STX3 was significantly affected in severe KC epithelium compared to control and mild KC epithelium (*P* < 0.05) (Fig. 3D).

### β-catenin localization and loss of barrier function

**Figure 4:**
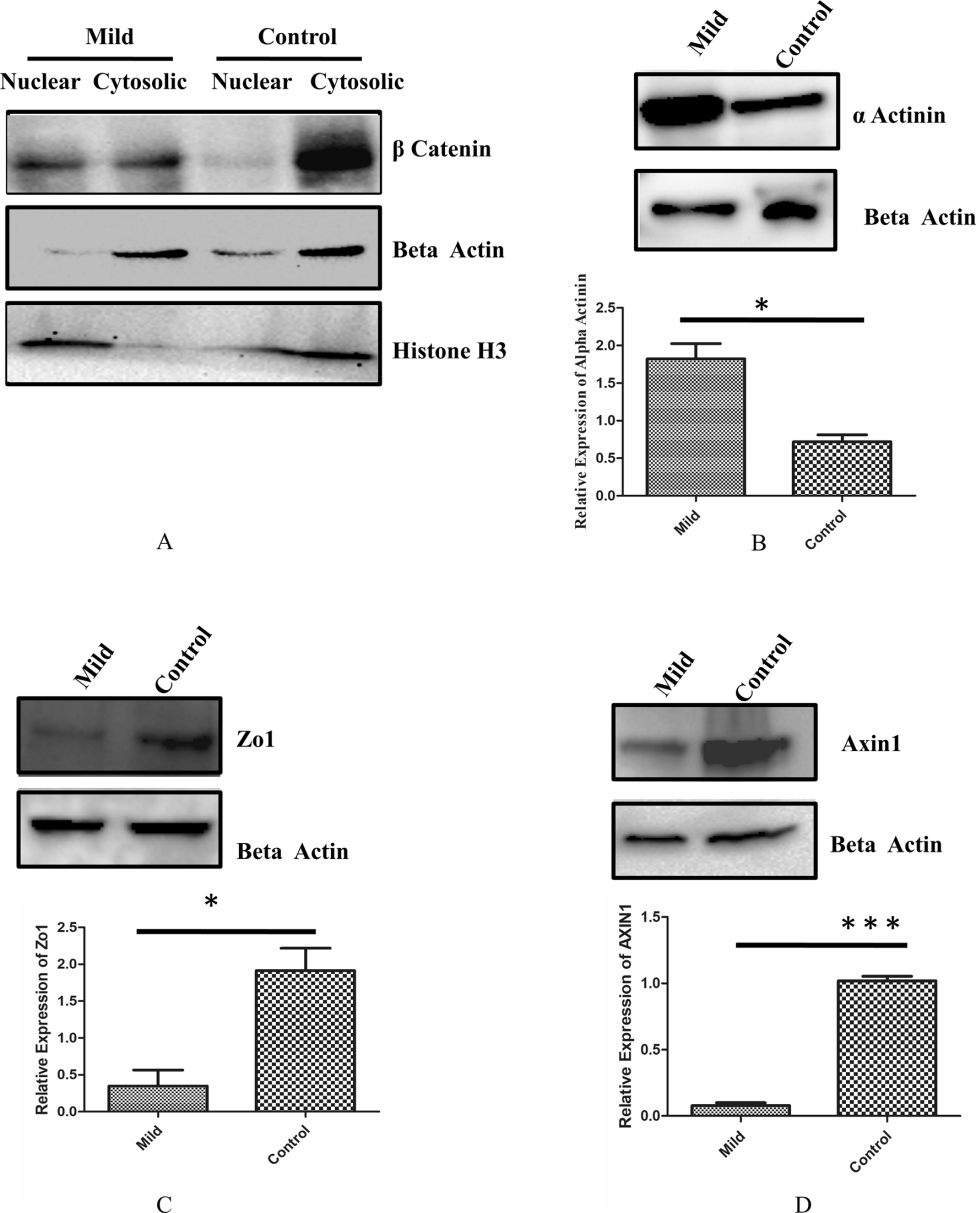
(**A**) Immunoblotting showing the Cytosolic and Nuclear fraction of β-catenin in control and mild KC epithelium (Full blot was cut in three halves for probing Histone H3,β catenin and beta actin Epiwhite merged image is represented in Fig S14) (**B**) Immunoblotting of α-actinin in control and mild keratoconic epithelium(Full blot was cut in two halves for probing α-actinin and beta actin image is represented in Fig S15) (n = 7) (**C**) Immunoblotting of ZO1 in control and mild keratoconic epithelium(n = 7) (Full blot was cut in two halves for probing ZO1 and beta actin Epiwhite merged image is represented in Fig S16) (**D**) Immunoblotting of Axin1 in control and mild keratoconic epithelium(n = 7)(Full blot was cut in two halves for probing Axin1 and beta actin image is represented in Fig S17).

To validate β-catenin localization (Fig. 1A), we performed cell fractionation of mild KC epithelium. The results showed that in mild KC epithelium, β-catenin localization is enriched in nuclear fraction (Fig. 4A). The loss of membrane bound β-catenin could be associated with loss of epithelial polarity and provide migratory nature to epithelium. Hence, we analysed the expression of α-actinin, which is known crosslinker for actin polymerization. Actin staining data (Fig S2) showed that the actin polymerization was altered. Significant upregulation of α-actinin in mild KC epithelium (*P* < 0.05) was observed suggesting that the KC epithelium is likely to be more migratory (Fig. 4B). The loss of membrane bound β-catenin known to affect the tight junction protein ZO1. In our data also, we found ZO1 to be significantly (*P* < 0.05) down regulated (Fig. 4C). Our immunofluorescence analysis of ZO1 in mild KC tissues showed less ZO1 staining compared to control (Fig S3). Further, the protein Axin1 which stabilises the β-catenin degradation complex in the cytosol^24^ was found to be significantly downregulated in mild KC corroborating with our data showing the nuclear localization of β-catenin (Fig. 4D). This data suggests that loss of membrane bound β-catenin induces the loss of epithelial integrity in KC epithelium.

### β-catenin Co-immunoprecipitation and analysis of its interacting partners using mass spectrometry

**Figure 5:**
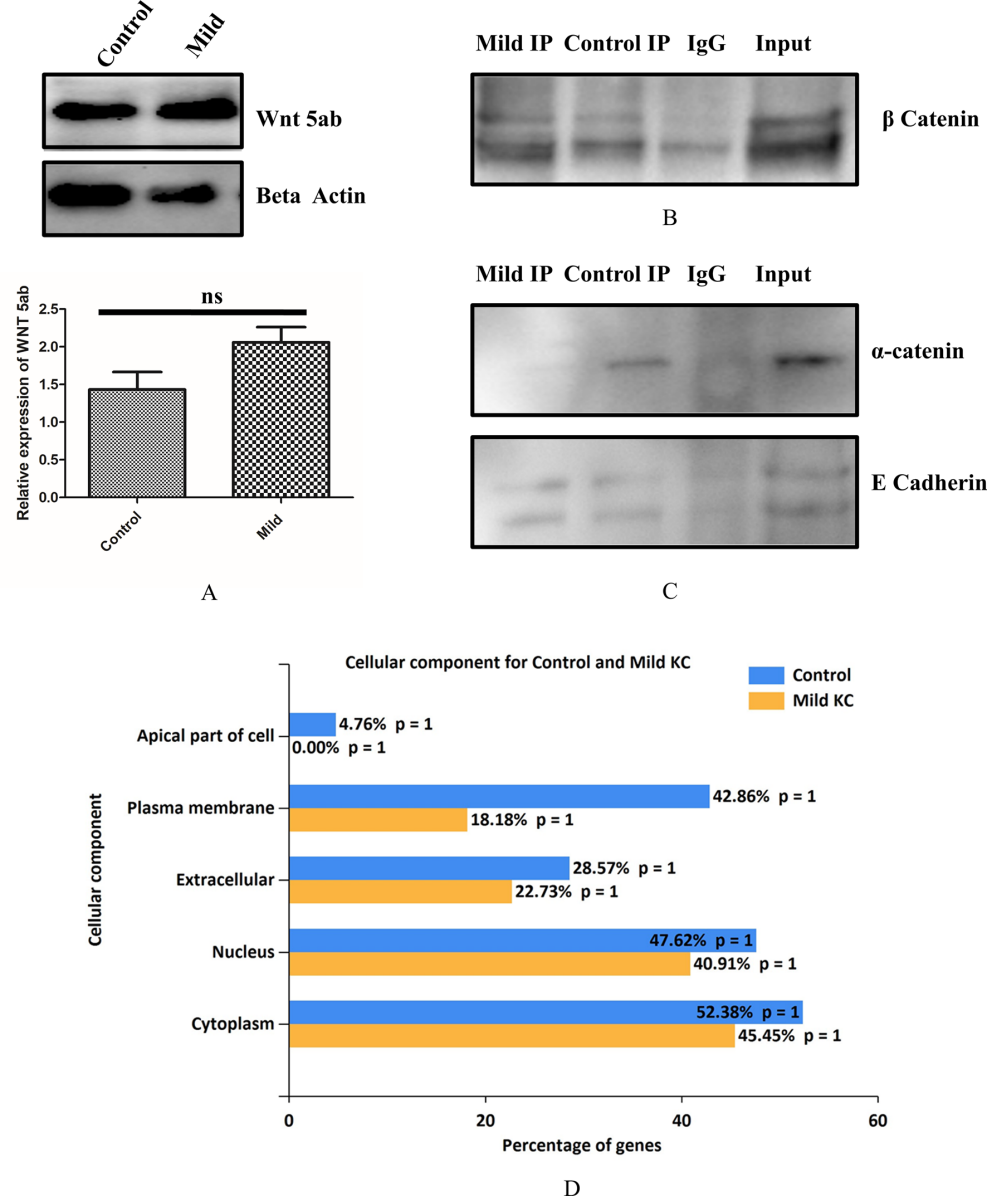
**(A)** Immunoblotting of Wnt5ab in control and mild KC epithelium (n = 7)**. (**Full blot was cut in two halves for probing Wnt5ab and beta actin image is represented in Fig S18) **(B&C)** Immunoblotting of β-catenin, E cadherin and α-catenin for the of β-catenin Co-IP complex in control and mild KC epithelium( β catenin CoIP blot was cut in to two halves for probing β catenin and beta actin. β catenin image has been shown in fig S19) (Full gel blot for E cadherin and alpha catenin is represented in fig S20 and S21) **(D)** Functional enrichment analysis of the localization of proteins which came as a hit in β-catenin Co-IP in control and mild KC epithelium.

We explored the role of Wnt signaling in KC, that mediates either nuclear localization or cytosolic β-catenin degradation by the proteasomal complex. In KC the expression of β-catenin was unaltered (Fig. 1B) indicating the lack of proteasomal degradation. Therefore, we next investigated the non-canonical Wnt signalling. We observed that there was no significant difference in the expression level of Wnt5ab protein in mild KC epithelium compared to control (Fig. 5A). However Axin1 was significantly downregulated in mild KC epithelium compared to control (Fig. 4C). The observed stabilization of β-catenin in the cytoplasm may not be due to altered Wnt signalling but could be due to the influence of other interacting proteins. Alternatively, the cytoplasmic pool of β-catenin which is destined for ubiquitination and proteasomal degradation can be stabilized by the presence of other interacting proteins in the diseased condition. This prompted us to perform an endogenous Co-IP of β-catenin in control and mild KC epithelium which further exhibited undegraded β-catenin, along with IgG control. The presence of β-catenin was observed in the co-immunoprecipitated product (Fig. 5B). Further, we looked for the known interacting proteins of β-catenin in this precipitate. β catenin interacts with cytoskeletal protein actin which is stabilized by α-catenin and E cadherin proteins. Hence, we investigated whether the loss of membrane bound β-catenin has any effect on these binding partners. Interestingly, the interaction between β catenin and E cadherin was intact whereas the interaction between β catenin and α catenin was lost in the mild KC epithelium (Fig. 5C).

The Co-IP was performed to capture the altered interacting proteins, of β-catenin in the mild KC. We then compared it to the protein interactions in the control epithelium where β catenin has membrane localization. The proteins were separated on SDS gel and tryptic digested in the gel. Mass spectrometry was performed as described in the experimental section to identify novel β-catenin interacting proteins. The interacting proteins were identified from the MS/MS spectra of the peptides (Fig S4, MS–MS spectra data) using Mascot server. Endogenous β-catenin from the tissues, the bait protein which was used for the pull-down assay, was detected in both control and KC tissue lysates. Endogenous Co-IP method contains proteins that are often non-specifically bound to the target protein. Hence, to distinguish these irrelevant background proteins, we devised a negative control using IgG antibody with the same concentration as that of β-catenin antibody. The proteins identified by mass spectrometry in the negative control (IgG immunoprecipitated from control tissues) were subtracted from our downstream data analysis (Table 2) when we compared the protein identifications from the control and the mild KC epithelium. The list of β-catenin interacting proteins in control and mild KC epithelium are listed in Tables 2 and 3. In control epithelium the interacting proteins of β-catenin are Histone Deacetylase 4, Vascular endothelial growth factor receptor1, Sox2 and RhoGTPase-activating protein 21. Some of these proteins are known interacting proteins of β-catenin in other cell lines. However, these proteins are novel identifications in corneal epithelium which are lost in mild KC epithelium. The novel interacting proteins observed in the control epithelium are Contactin-1, Pappalysin-1, Mimecan which are mostly extracellular matrix regulating and cell surface interacting proteins. The novel β-catenin interacting proteins identified in mild KC epithelium were StAR-related lipid transfer protein 3, Dynamin-1-like protein, Cardiotrophin-1, Musculin, Basal cell adhesion molecule and Protocadherin Fat 1. The known interacting proteins identified are Collagen alpha-1 chain and Laminin subunit alpha-4. Tenascin C was identified in both control and mild KC epithelium.

**Table 2:** β-Catenin interacting partners from control corneal epithelium using Co-IP/MS approach.

Protein name	Gene name	Molecular weight (kDa)	UniProt ID	Score
Histone Deacetylase 4	HDAC4	140	P56524	66
Vascular Endothelial growth factor receptor 1	FLT1	180	P17948	47
AH receptor-interacting protein	AIP	38	O00170	45
Ubiquitin-conjugating enzyme E2	UBE2D2	17	P62837	40
Bromodomain-containing protein	BRD4	200	O60885	40
SOX-2	SOX2	35	P48431	39
Focadhesin	FOCAD	200	Q5VW36	39
Alpha-enolase	ENO1	47	P06733	37
Sialic acid binding IgG like lectin 11	SIGLEC11	76	Q96RL6	36
Interleukin-18 receptor	IL18RAP	68	O95256	35
Contactin-1	CNTN1	113	Q12860	34
Pappalysin-1	PAPPA	180	Q13219	34
Carbonic anhydrase 2	CA2	28	P00918	33
RhoGTPase-activating protein	ARHGAP21	217	Q5T5U3	32
Epithelial-stromal interaction protein 1	EPISTI1	37	Q96J88	32
Krueppel-like factor 10	KLF10	52	Q13118	32
CXC chemokine receptor type 6	CXCR6	39	O00574	32
Tenascin C	TNC	225	Q99857	32
Chondroitin sulfate N-acetylgalactosaminyltransferase	CSGALNACT1	61	Q8TDX6	31
G1/S-specific cyclin-E2	CCNE2	48	O96020	30
Peroxisomal membrane protein	PEX11A	28	O75192	30
Retinoic acid-induced protein	GPRC5A	40	Q8NFJ5	30
Mimecan	OGN	34	P20774	30
Plakophilin-1	PKP1	83	Q13835	30
Protein phosphatase 1D	PPM1D	67	O15297	30
Tetkin4	TEKT4	51	Q8WW24	30

**Table 3:** β-Catenin Interacting Partners from Mild KC Corneal Epithelium using Co-IP/MS approach.

Protein name	Gene name	Molecular Wt. (kDa)	UniProt ID	Score
StAR-related lipid transfer protein 9	STARD3	50	Q14849	51
Protocadherin Fat 3	FAT1	500	Q14517	50
Collagen alpha-1 chain	COL1A1	220	P02452	46
Dynamin-1-like protein	DNM1L	81	O00429	46
Tenascin C	TNC	225	Q99857	44
Laminin subunit alpha-4	LAMA4	202	Q16363	43
Cilia- and flagella-associated protein 47	CFAP47	361	Q6ZTR5	42
Serine/threonine-protein kinase RIO2	RIOK2	63	Q9BVS4	42
Sperm-associated antigen	SPAG5	134	Q96R06	41
DNA-directed RNA polymerase III subunit	POLR3A	156	O14802	40
A disintegrin and metalloproteinase with thrombospondin motif	ADAMTS5	75	Q9UNA0	36
Tumor necrosis factor alpha-induced protein	TNFAIP3	82	P21580	36
Anoctamin 6	ANO6	98	Q4KMQ2	35
BBSome-interacting protein	BBIP1	10	A8MTZ0	35
Cardiotrophin-1	CTF1	21	Q16619	35
Keratin, type I cuticular	KRT81	56	Q14533	35
Probable tubulin polyglutamylase TTLL9	TTLL9	51	Q3SXZ7	34
Activity-regulated cytoskeleton-associated protein	ARC	27	Q7LC44	33
Musculin	MSC	22	O60682	32
Aurora kinase A-interacting	AURKAIP1	22	Q9NWT8	32
Collagen alpha1 XVI	COL16A1	157	Q07092	32
Serine/threonine-protein kinase STK 11	STK11	55	Q15831	32
Basal cell adhesion molecule	BCAM	67	P50895	31
Oxysterol binding protein 1	OSBP	89	P22059	30
RNA polymerase 2 elongation factor	ELL3	45	Q9HB65	30

Bioinformatics functional enrichment analysis in the control epithelium identified proteins to be localized (42.86%) in plasma membrane whereas in mild KC, only 18% of the identified proteins were localized to plasma membrane (Fig. 5D).

### Validation of the Co-IP proteins and β-catenin response to substrate stiffness in control epithelium

**Figure 6:**
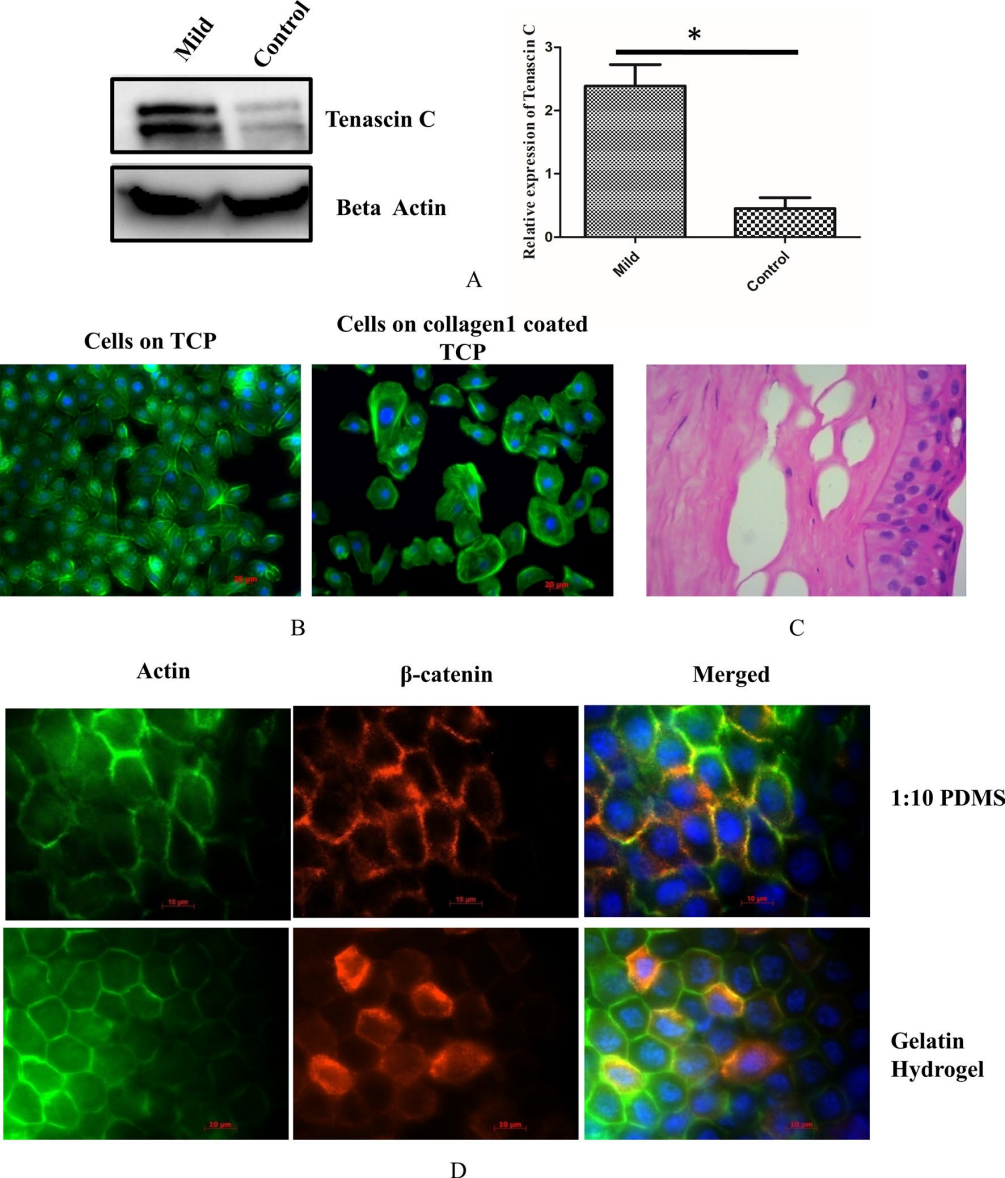
**(A)**Immunoblotting of Tenascin C in control and mild KC epithelium(n = 3) (Full blot was cut in two halves for probing Tenascin C and beta actin image is represented in Fig S22) **(B)** Phalloidin staining of corneal epithelial cells on plate with and without collagen coating Scale bar-20 µM **(C)** Histological section of KC from patients undergoing penetrating Keratoplasty (n = 7) **(D)** β-catenin localization of epithelial graft cultured on 1:10 PDMS and Gelatin Hydrogel Scale bar-10 µM.

Tenascin C was identified by mass spectrometry and was further validated by Western blotting in both control and mild KC tissue. We observed Tenascin C expression in both control and KC tissues. Furthermore, we observed upregulation of Tenascin C in mild KC epithelium compared to the control (*P* < 0.05) (Fig. 6A). Next, we studied a protein that was identified exclusively in KC. We identified collagen alpha chain 1 protein exclusively in KC epithelium. This is possibly explained by the fact that a breakage of Bowman’s layer exposes the epithelial cells to collagens present exclusively in the stroma. Therefore, we coated the TCP with collagen 1 and studied the actin reorganization compared to the non-collagen coated plates. We observed distinct actin staining in corneal epithelial cells grown on collagen matrix compared to the non-coated one (Fig. 6B) indicating the effect of modification of ECM on corneal epithelial cells. Similar alterations in ECM could contribute to the pathogenesis of KC which shows predominant actin staining compared to control (Fig. 3A).

**Figure 7:**
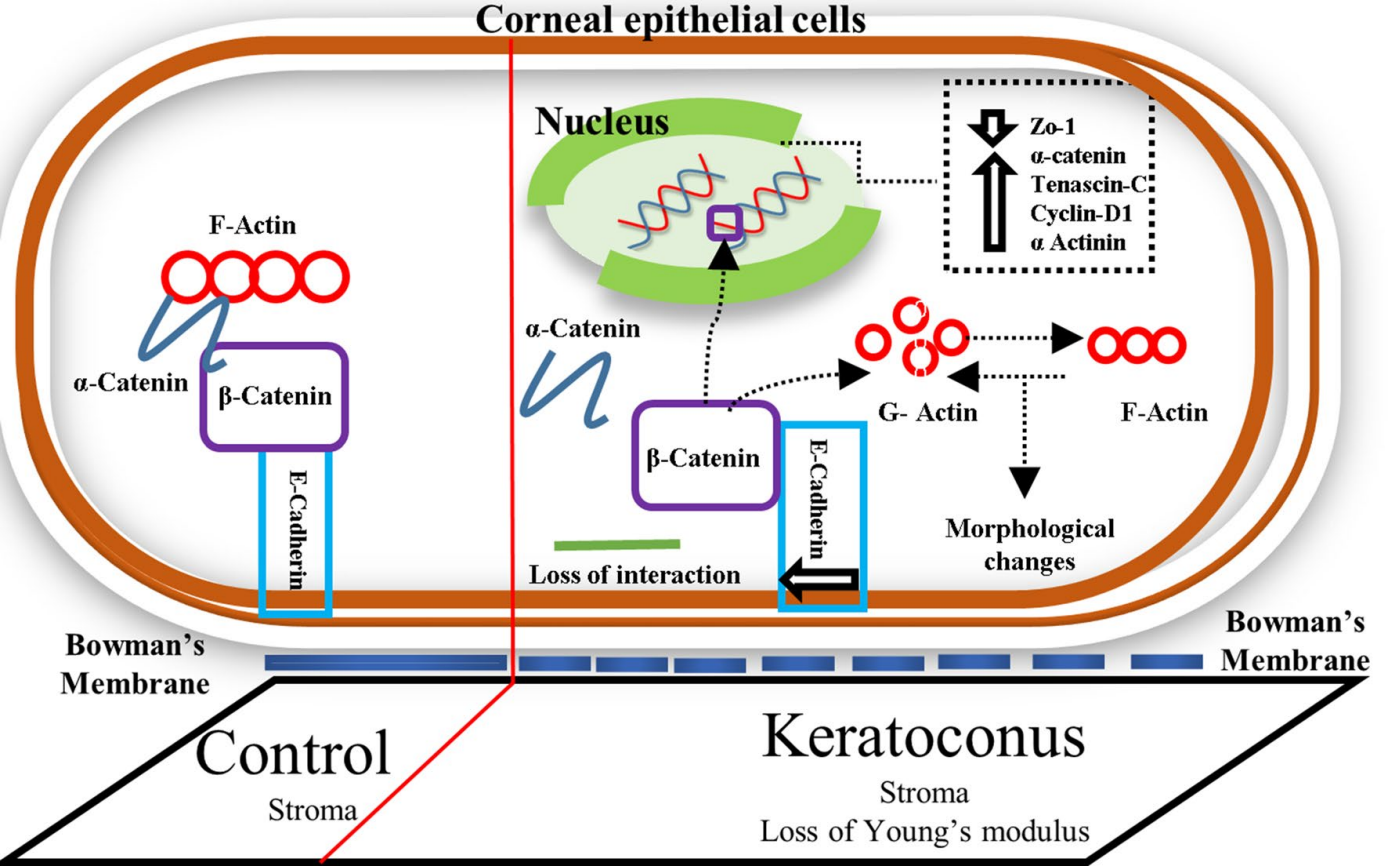
Pictorial representation of β-catenin dependent mechanotranduction in corneal epithelium.

Histopathology data from our archive study also confirmed the Bowman’s layer breakage in KC (Fig. 6C) sample. To validate this hypothesis, we prepared 2 different stiffness matrix such as PDMS (35 kPa) and gelatin hydrogel 7 kPa (Fig S5). As a control, we measured the elastic modulus of normal cadaveric stroma and found to be 25 ± 5 kPa which is in agreement to the published reports^25^. β-catenin localization on epithelial graft grown on PDMS showed membrane localization whereas on gelatin hydrogel it showed both membrane and cytoplasmic localization similar to the epithelium of mild KC (Fig. 6D). Hence, our data suggests that, β-catenin in corneal epithelium responds distinctly to changes in extracellular matrix stiffness. The events leading to β-catenin dependent mechanotransduction have been graphically represented in Fig. 7.

## Discussion

KC is a bilateral progressive ectatic corneal disease resulting in profound visual impairment. The exact cause and site of origin of the disease remain uncertain. Histopathological changes have been documented in all layers of the cornea except the endothelium. There are several recent reports concluding that the corneal epithelium remodels in response to underlying stromal irregularities^26,27^ and even suggest that corneal epithelial thickness mapping may be useful in detecting subclinical and early Keratoconus^28,29^. Histological & miRNA studies demonstrate structural & biological changes in corneal epithelium of KC^30^.

Substrate stiffness is known to affect cellular behaviour^31^. However, the mechanism underlying mechanosensing in the cornea and the exact signalling pathways involved in the changes of corneal epithelial cells morphology & behaviour have not been elucidated yet. β-catenin is a multifunctional protein and its subcellular localization plays a pivotal role in cellular signalling. The membrane bound β-catenin is associated with adhesion function whereas, the nuclear form plays a role in transcriptional regulation^32^. The cytoplasmic pool of β-catenin is highly unstable in the absence of Wnt signal^33^. At the membrane, β-catenin forms complex with E-cadherin and α-catenin which in turn maintain the structural integrity of the epithelial cells. The localization of β-catenin in central corneal epithelium of healthy eyes has been reported to be membrane bound^21^. Recent studies on KC corneal epithelium using RNA sequencing have highlighted that TGFβ, Hippo signalling and Wnt signalling pathway are dysregulated. Non canonical Wnt such as Wnt4, Wnt7A and Wnt7B has been reported in KC epithelium^8^. However, to the best of our knowledge no detailed study has been performed on the role of β-catenin localization and its interaction in KC epithelial cells.

Our results (immunofluorescence, immunohistochemistry & cellular fractionation) confirmed that in KC epithelium, the localization of β-catenin has been altered and is cytosolic and nuclear. However, there is no difference in the total β-catenin protein expression. On further investigation of the interacting partners of membrane bound β-catenin, we observed significant down regulation of E-cadherin and α-catenin expression in KC corneal epithelium. The role of E-cadherin could  possibly be compensated by increased Pan cadherin expression. Additionally, membrane bound E-cadherin was lost indicating the loss of cell–cell adhesion. To maintain a polarized epithelium, actin cytoskeleton co-operates with E-cadherin- and integrin-based cell–cell or cell–matrix adhesions^34^.α-actinin, a major actin filament crosslinker was found to be significantly upregulated in mild KC epithelium. α-actinin upregulation has also been reported in human breast cancer and mammary epithelial cells where it promotes cell migration and induces disorganized acini like structures^35^. The altered KC corneal epithelial structure resulted in the loss of polarity indicated by reduced syntaxin 3 expression. Furthermore, loss of the epithelial polarity could also lead to altered tight junction structure and function, ZO1 was significantly downregulated in KC epithelium. The loss of both E-cadherin and ZO1 could be associated with changes in morphology of KC epithelium which has altered cellular adhesions^15^. The altered cell–cell adhesion might have caused different stress fibers localization in the KC epithelium. In the mild KC epithelium, stress fibers were located mostly at the membrane of the cells whereas in severe KC epithelium the stress fibers were found across the cells. This could be due to changes in the actin polymerization. The alteration in stress fiber formation and actin polymerization are regulated by RhoA, and Rac^36^. We also found RhoA, and Rac123 to be altered in mild KC. The observation from our studies suggested that loss of membrane bound β-catenin could be reason for the loss of adherence junction proteins which in turn alters the cellular morphology of KC epithelium. Consistent with our studies, an altered cellular morphology of reduced basal epithelial cell density in moderate and severe keratoconus and greater average basal epithelial cell diameter were observed in keratoconic patients^15,37^.

The localization of β-catenin is also dependent on Wnt signalling^38^. However, this protein may not be directly involved in disease progression of KC as β-catenin was unaltered in the diseased condition. β-catenin is a central molecule in Wnt signaling pathway and the transcriptional activity of β-catenin is also modulated by direct regulation of its subcellular localization by a variety of interacting partners^39^. In mild KC epithelium in the absence of the Wnt signaling, the loss of interaction between β-catenin and α-catenin might lead to lowered ubiquitylation and stabilization of β-catenin. Similarly, the ubiquitylation of native β-catenin was strongly inhibited in α-catenin depleted cells leading to stabilization of the protein^40^. Additionally, the known interacting proteins Collagen alpha-1 chain and Laminin subunit alpha-4 could also alter the localization of β-catenin. Furthermore, in response to collagen 1 coating, actin reorganization was altered in corneal epithelial cells. β-catenin localization was altered in response to matrix stiffness (PDMS to gelatin hydrogel) mimicking KC. Additionally, Tenascin C was found in both control and mild KC epithelium, it was upregulated in KC due to the presence of the β-catenin in the cytosol and possibly in nucleus. Tenascin C has an antiadhesive activity and has been reported to be regulated by β-catenin^41^. Therefore, we summarize that β-catenin might sense the altered stiffness in KC when the Bowman’s layer is ruptured. It is established that various layers of cornea exhibit different elastic moduli^25^ and as the disease progresses the epithelium is exposed to matrix of different elastic moduli.

In summary, our study revealed distinct changes in the localization of β-catenin in the corneal epithelium of patients with all clinical grades of Keratoconus. These changes appear to be directly influenced by changes in the elastic modulus of the substrate, and were associated with the downregulation of E cadherin and α-catenin that were also observed. The total expression of β-catenin was unchanged, suggesting a loss of interaction between β-catenin and α-catenin as demonstrated by the co-immunoprecipitation studies. Additionally, there was loss of ZO-1 tight junction protein and upregulation of α-actinin. Overall, these changes may contribute to the altered structure and polarity of the corneal epithelial cells and may be expected to make them less adhesive and more migratory in nature. Based on the consistency of our observations, we propose that β-catenin could be considered as a potential mechanotransducer in the corneal epithelium .

## Materials and methods

### Collection of tissue samples from patients undergoing photorefractive corrections and collagen crosslinking

This study, which conformed to the tenets of the Declaration of Helsinki, was reviewed by the local ethics committee and approved by the institutional review board of Vision Research Foundation, Sankara Nethralaya, Chennai India (Ethics No. 659-2018-P).The tissues used for the study were taken from patients after obtaining their signature and informed consent.

All study subjects were clinically examined at the department of Cornea, Medical Research Foundation, Chennai, India and the clinical details are presented in Table 1. The epithelium of patients undergoing collagen crosslinking for documented progressive KC was used for the study. The epithelium of normal myopic patients undergoing photorefractive keratectomy (PRK) for corrections of their refractive error served as normal control (Ethics No. 489–2015-P). The severity of KC was graded using Amsler-Krumeich classification^42^. The clinician used an epikeratome to remove the epithelium as a single sheet while performing Photorefractive keratectomy (PRK). Care was taken not subject it to any mechanical stress. The control tissues to be used for cell culture were collected in Defined Keratinocyte-Serum Free medium (SFM) (Thermofisher Cat.No-10744019) and were processed immediately after the collection. However, the control and the KC epithelium tissues which were subjected for immunoblotting, Tissue immunofluorescence and Co-immunoprecipitation (Co-IP) was collected in DMEM F-12(Gibco) medium with sodium Pyruvate (Sigma) and transported to the laboratory. Concurrently, Polydimethylsiloxane (PDMS) (Sylgard 184 Silicone Elastomer kit) and gelatin (Bovine skin Type B Sigma) hydrogel of different elastic modulus were prepared as described to serve as substrate for culture of epithelial tissues from normal control^43^.

### Western blotting

Tissue collected was dissolved in RIPA (Radioimmunoprecipitation assay) buffer (EMD Millipore Cat No-20-188), sonicated in ice for 15 s, and centrifuged at 13,000×*g* for 10 min at 4 °C. The supernatant was collected, Proteins were estimated using BCA protein assay kit (Thermo Fisher Cat No-23227). For Western blot, equal concentration of tissue lysates were loaded onto the gel and separated on a sodium dodecyl sulphate polyacrylamide gel (SDS-PAGE) at 100 V in electrophoresis buffer (25 mM Tris, 190 mM Glycine and 0.1% SDS). The proteins were separated and transferred to PVDF membrane (GE Healthcare Cat No- 10,600,023) using semi-dry transblot apparatus (Hoefer) at 1.50 mA/cm^2^. The membrane was then blocked for 1 h at 25 °C in 5% (w/v) non-fat dry milk powder (NFDM) in TBST (20 mM Tris–HCl pH 7.5, 150 mM NaCl and 0.1% Tween 20). It was washed with TBST, incubated at 4 °C overnight with the following antibodies :E-cadherin, Pan cadherin, α-catenin, β-Catenin, from Cadherin-Catenin antibody sampler kit (Cell signaling and Technology Cat No-9961) β-Catenin for Co-IP (Cell Signaling Technology Cat No-9562S), β-Catenin for immunoblotting (Abcam-ab22656) ZO1, Wnt5ab, RhoA, Rac1/2/3, α-Actinin (Cell signaling and Technology Cat No-13663, 2520 T, 21,170, 2462, 3134) β-actin, Tenascin C and Cyclin D1 (Santa cruz Cat No-sc47778, sc-20932, sc 246), Syntaxin 3 (Synaptic system 110,032). Each antibody was 1000-fold diluted in either 5% (w/v) BSA (Hi Media Cat No-MBO3) or NFDM in TBST. After overnight incubation, the membrane was washed thrice for 5 min with TBST and further incubated in corresponding HRP-conjugated Anti-rabbit and Anti-mouse secondary antibody). The secondary antibodies used were diluted to 10,000- fold in 5% NFDM (w/v) in TBST. After incubation, the membrane was again washed thrice for 5 min with TBST. HRP activity was detected using HRP substrate (Bio-Rad Cat No-1705061) in Bio-Rad Gel Documentation system (Protein Simple).

### Tissue immunofluorescence

Tissues collected from patients were fixed in 4% Paraformaldehyde and washed with Phosphate buffered saline (PBS).Tissues were permeabilized with 0.5% triton-100 followed by PBS wash. The tissues were then blocked with 1%BSA prior to overnight incubation with primary Antibody. The detection was done using Cy3.5 secondary antibody and counterstained with Hoechest (Thermo fisher Cat No-33342).Further actin fibers of the tissues were stained with 100 nM of Phalloidin-488 (Cytoskeleton, INC. Cat No-PHDG1) after permeabilization. The tissues were incubated for 45 min in dark and Counter-stained with Hoechst**.** The tissues were imaged using Zeiss Microscope using 2 different channels.

### Immunohistochemistry

Formalin-fixed paraffin-embedded (FFPE) tissue blocks of control and KC epithelium were made and processed for immunohistochemical analysis. Immunohistochemistry (IHC) was performed on Bench Mark GX (Ventana Roche) Automated devices for the Bio-SB antibody at M/s S.M Surgipath, Chennai, using β-Catenin (Abcam-ab22656) and YAP (CST-14074) antibodies. β catenin protein was visualized with HRP conjugated anti mouse antibody (Sigma Aldrich). The peroxidase was detected with 3,3′ diaminobenzidine (Sigma Aldrich) each section was counter stained with Mayer’s Haematoxylin (sigma Aldrich). All stained sections were analysed under a light microscope (Eclipse Ni-**U** Upright Microscope from Nikon Instruments Inc).

### Co-immunoprecipitation followed by Matrix assisted laser desorption/ ionization(MALDI) time-of-flight (TOF)

Control tissues and mild KC tissues were pooled separately after collecting from patients for performing co-immunoprecipitation(Co-IP). Endogenous co-immunoprecipitation(Co-IP) was performed to identify the novel interacting partner of β-catenin. For performing co-immunoprecipitation(Co-IP), around 500 μg of protein lysate was required as reported earlier. Hence, we pooled lysates from 4 Control samples and designated as C1. Similarly, C2 and C3 lysates were prepared. For KC also, lysates from 4 mild KC samples were pooled and designated as K1. Similarly K2 and K3 lysates were also prepared. Tissue lysates were prepared using RIPA buffer. 400 μg lysates, were precleared using 40 μl of Protein-A Magnetic beads (New England Biolabs Cat No-S1425S) to remove non-specific components from the lysate. After preclearing 4 μg of β-catenin antibody (Cell signaling and Technology, Cat No-9562S) and IgG antibody(Cell signaling and Technology Cat No-2729 s) were incubated overnight at 4 °C. Further, 40 μl of Protein A/G Magnetic bead suspension were added and incubated with precleared lysate, agitated for 1 h at 4 °C. Magnetic field was then applied to pull the beads to the side of the tube and 3 washes were performed using Wash buffer. Further the bead pellet was suspended in 5X sample loading buffer.

### SDS page and in-gel digestion

The immunoprecipitated product was loaded on 10% SDS Polyacrylamide gel. The electrophoresis of the sample was carried out using Tris–Glycine buffer at 100 V. After the run was completed the proteins were stained with Coomassie blue stain (Sigma Cat No-G1041).The gel band from IgG Lane, β-catenin IP from control tissue and β-catenin IP from mild KC tissue were excised into 1 mm^3^. The gel was washed with washing solution (50% acetonitrile, 50 mM ammonium bicarbonate) until the Coomassie die was completely removed and incubated at room temperature for 15 min with gentle agitation. The gel was then dehydrated using 100% acetonitrile(ACN). Then ACN was removed, and the dry gel was kept at room temperature for 10–20 min in a vacuum evaporator. The gel was rehydrated for 30 min at 56 °C using reduction solution (10 mM DTT, 100 mM ammonium bicarbonate). Further, alkylating solution (50 mM iodoacetamide, 100 mM ammonium bicarbonate) was added and incubated for 30 min in the dark at room temperature. After sequential washing with 25 mM NH_4_HCO_3_, 25 mM NH_4_HCO_3_/50%ACN, and 100% ACN, gel pieces were dried and rehydrated with 12.5 ng/mL trypsin (Promega, Madison, WI) solution in 25 mM ammonium bicarbonate on ice for 30 min. The digestion was continued at 37 °C overnight. The tryptic peptides were extracted from gel pieces with extraction solution (60% ACN, 0.1% TFA) and sonicated in ultrasonic water bath for 10 min. Further extracted peptides were resuspended in 50% acetonitrile, 0.1% TFA. The samples were spin down and spotted 0.5µL volume on alpha-cyano-4-hydroxycinnamic acid matrix (10 mg/mL in 50% ACN, 0.1% TFA) of MALDI plate. The spots were allowed to dry and further plate was loaded into Voyager.

### Scanning electron microscopy

Scanning electron microscopy (FEG-SEM Quanta 400 instrument; FEI), was used to analyse the morphology of epithelial tissue collected after surgery. The tissues were fixed with 3.7% glutaraldehyde (Sigma Cat No- G5882) in PBS for 15 min in aluminium-coated coverslip. After washing twice with PBS, the fixed tissues were dehydrated with an ascending sequence of ethanol (40%, 60%, 80%, 96–98%). After evaporation of ethanol, the samples were left to dry at room temperature for 24 h on a glass substrate, and then analyzed by SEM after gold–palladium sputtering .

### Preparation and characterization of poly dimethyl siloxane gel and gelatin hydrogel

Polydimethylsiloxane (PDMS) gel was prepared using polymer and curing agent in a ratio of 1:10 and incubated at a temperature of 80 °C for 24 h in 24 well plate. Further, the PDMS were coated with type-1collagen (Sigma Cat No -C4243).Hydrogels were prepared using type B gelatin (Sigma G9391). 50 mg/ml of gelatin concentration with 0.8 µg/mL of glutaraldehyde as crosslinker were used for preparing hydrogel and incubated at 4 °C for 4 h for gelation and cross-linking. The cross-linked gelatin hydrogels were immersed in a 50 mM glycine aqueous solution under agitation for 1 h to block the residual aldehyde groups of glutaraldehyde, followed by two washes with double-distilled water for 1 h^43^.

The stiffness of the hydrogels were measured using Microsquischer (M/s.Cell Scale) instrument. The compressive method was employed using 3X3mm plate bound to microbeam. Different forces (500 µN, 1000 µN, 1500 µN) were used for obtaining the force Vs time and force Vs displacement curves. To measure the stiffness, stress (Force/Area) and % strain(tip displacement) were calculated. The graph of stress vs strain was plotted, and linear region was considered to calculate the slope to find the young’s modulus. The rheology (young’s modulus) of hard PDMS gel was measured using MCR301 rheometer (Anton Paar). As a positive control, we used cadaveric stroma along with PDMS. Dynamic oscillatory strain sweep experiments were completed on the PDMS/Cadaveric stroma to find the limit of viscoelastic property. The sample size of 1 cm radius was used between the compressive plates. The size and thickness of the sample was maintained. The dynamic time sweep was conducted at the frequency of 1 rad s^−1^ and the strain of 0.1% at 25 °C. G′ is the storage modulus for measuring the gel-like behaviour of a system, whereas G″ is the loss modulus for measuring the sol like behaviour of the system.

### Culturing of primary human corneal Epithelial cells on different matrix

Corneal epithelial cells were cultured in Defined Keratinocyte-Serum Free medium (SFM) (Thermofisher Cat.No-10744019).The tissues collected from control patients was cultivated on type 1 collagen coated 1:10 Polydimethoxy siloxane (PDMS) of desired elastic modulus and 50 mg/ml of Gelatin Hydrogel in Defined Keratinocyte-SFM medium (Thermofisher Cat.No-10744019).Adherence of the tissues to culture plate was assured by a gravitational force from viscoelastic solution added on top of the tissues (HEALONOVD, Abbott Medical Optics, USA) as reported earlier^44,45^. After 24 h of incubation, tissues were fixed and immunofluorescence imaging was performed.

### Hematoxylin and Eosin staining

Corneal section was deparaffinized in Xylene and then rehydrated in 100% and 95% ethanol. After washing, it was stained with Hematoxylin (HI Media GRM 236). The section was differentiated with 1% HCl in 70% ethanol, then washed with water, followed by blueing with weak ammonia. The slide was stained with eosin and then dehydrated slides were mounted.

### Statistical methods

The corneal epithelium was collected from documented KC patients. All experiments were performed on at least 7 independent patients samples. In the manuscripts ‘n’ represents individual patient samples. The statistical significance of the differences was analysed by one-way analysis of variance (ANOVA) followed by Bonferroni Post-hoc test when comparisons were done between control, mild, moderate and severe KC using Graph Pad software for the samples mentioned in the Figs. 1, 2 and 3. Paired student “t” test were performed for samples mentioned in Figs. 4, 5 and 6 when compared between control and mild KC. Asterisks *, **, and *** denote a significance with *p* Values < 0.05, 0.01, and 0.001 respectively.

## Supplementary information

Supplementary Information.
