# Supplementary material for: Deciphering the mechanoresponsive role of β-catenin in keratoconus epithelium
Source: Sci Rep. 2020 Dec 7;10:21382. doi: 10.1038/s41598-020-77138-3 (PMC7721701; doi:10.1038/s41598-020-77138-3)
Supplement: Supplementary file 1 — Supplementary Information. [file 41598_2020_77138_MOESM1_ESM.pdf]

Deciphering the mechanoresponsive role of β-catenin in Keratoconus epithelium

Chatterjee Amit [1,2], Prema Padmanabhan[3], Janakiraman Narayanan[#1]

[1] Department of Nanobiotechnology, Vision Research Foundation, Sankara Nethralaya campus, Chennai, Tamil Nadu, India

[2] School of Chemical and Biotechnology, SASTRA, Deemed University, Tanjore, Tamil Nadu, India

[3] Department of Cornea, Medical Research Foundation, Sankara Nethralaya campus, Chennai, Tamil Nadu, India

Running Title: β-catenin mechanotransduction in kerataconus

Correspondence: [#]Dr.Janakiraman Narayanan, Department of Nanobiotechnology, KNBIRVO Block, Vision Research Foundation, Sankara Nethralaya campus, 18/41, College road Nungambakkam, Chennai, Tamil Nadu, India 600006, Tel-+91-44-28271616, (Ext) 1358, Fax-+91-44-28254180, Email: drjrn15@gmail.com

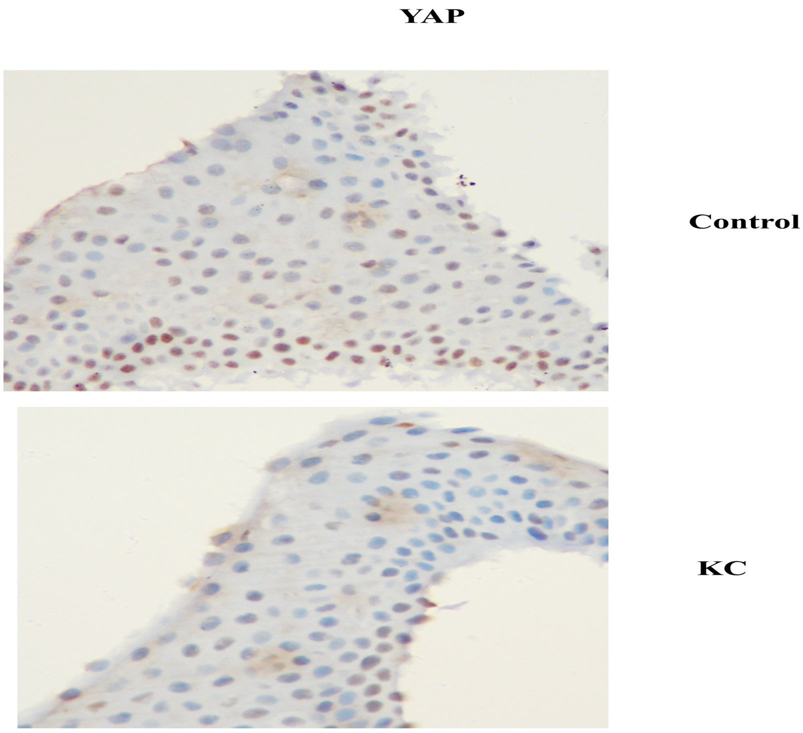

Supplementary S1: Immunohistochemistry YAP in KC epithelium

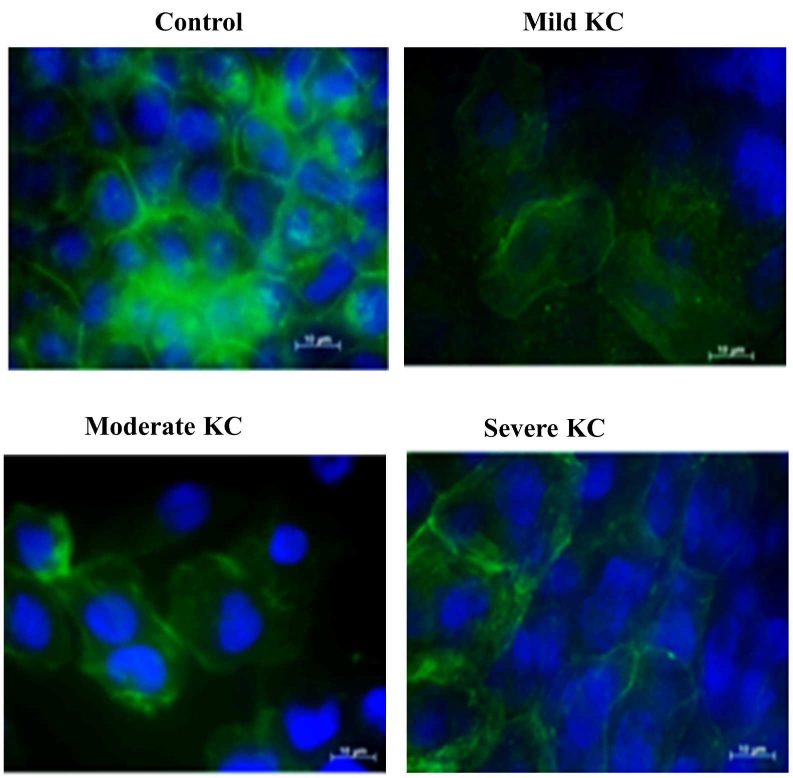

S2

Supplementary S2 Actin staining of control and KC tissues (mild, moderate, and severe)
Scale bar-10 μM

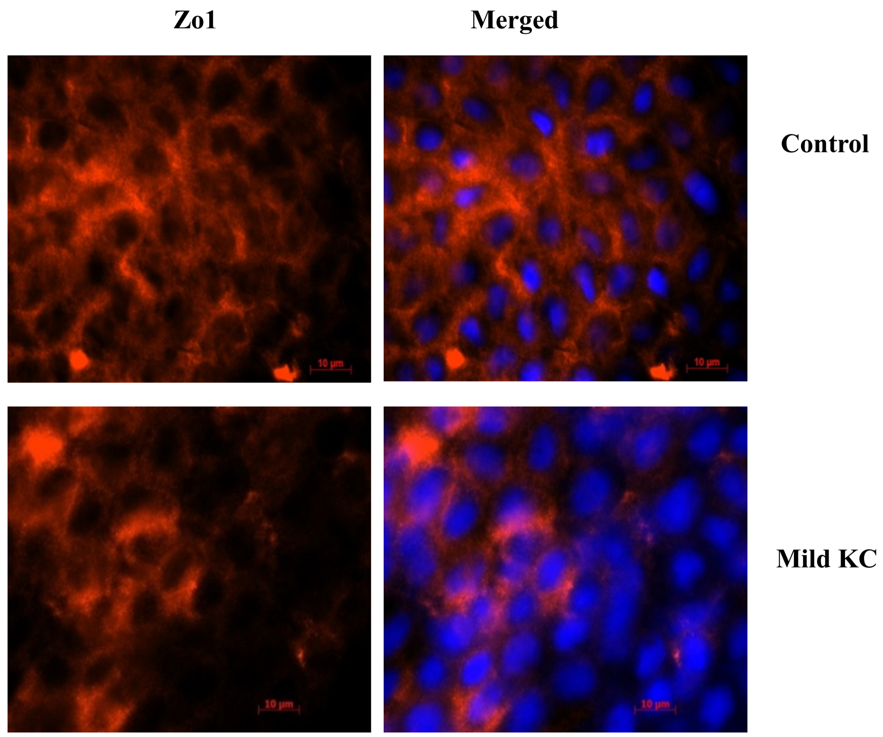

S3

Supplementary S3: ZO1 staining of control and mild KC Scale bar-10 μM

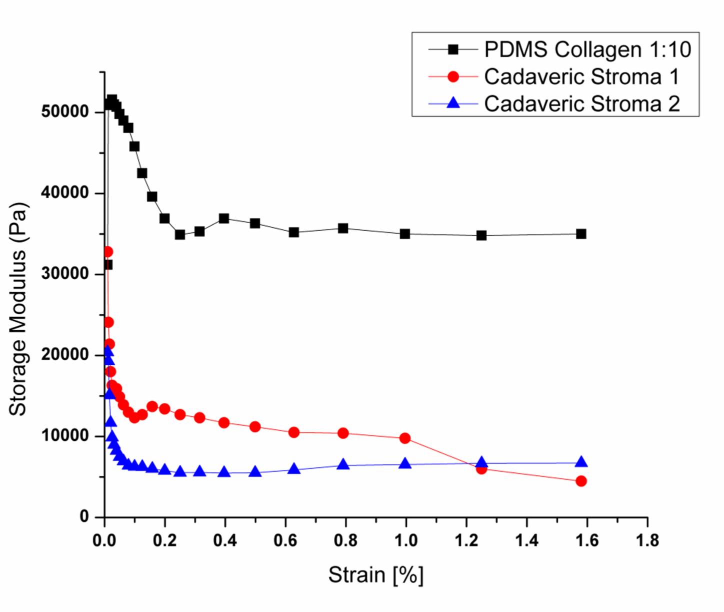

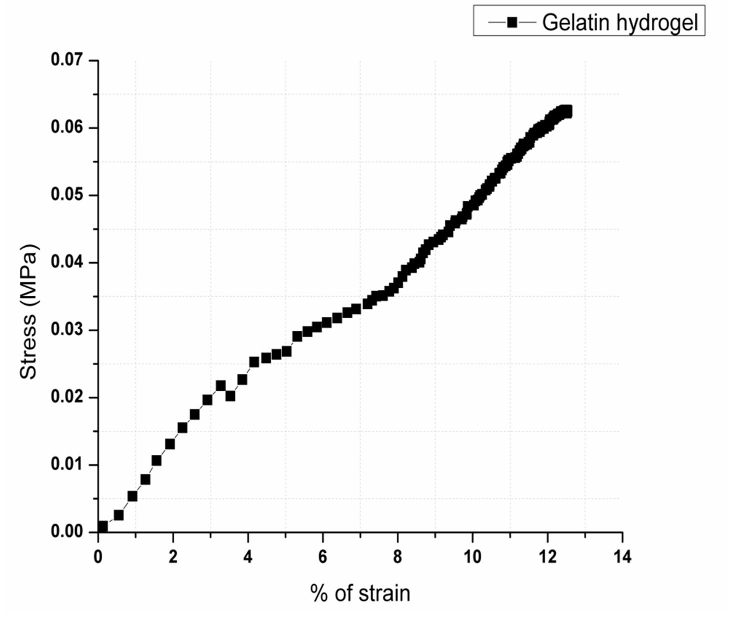

S5

Supplementary S5 : Elastic modulus measurement of PDMS substrate, cadaveric stroma and Gelatin hydroel

Title: Deciphering the mechanoresponsive role of β-catenin in Keratoconus epithelium

Chatterjee Amit [1,2], Prema Padmanabhan[3], Janakiraman Narayanan[#1]

[1] Department of Nanobiotechnology, Vision Research Foundation, Sankara Nethralaya campus, Chennai, Tamil Nadu, India

[2] School of Chemical and Biotechnology, SASTRA Deemed University, Tanjore, Tamil Nadu, India

[3] Department of Cornea, Medical Research Foundation, Sankara Nethralaya campus, Chennai, Tamil Nadu, India

Running Title: β-catenin mechanotransduction in kerataconus

Correspondence: [#]Dr.Janakiraman Narayanan, Department of Nanobiotechnology, KNBIRVO Block, Vision Research Foundation, Sankara Nethralaya campus, 18/41, College road Nungambakkam, Chennai, Tamil Nadu, India 600006, Tel-+91-44-28271616, (Ext) 1358, Fax-+91-44-28254180, Email: drjrn15@gmail.com

Sample Legend

Sample1-IgG Epilasik 225-100

Sample2-IgG Epilasik 100-25

Sample 3-IgG 25 below

Sample 4-Beta catenin Ip EpiLasik 225-100

Sample 5-Beta cateninIP 100-25

Sample 6-Beta catenin Ip 25 below

Sample 7- Beta catenin Ip Mild KC 225-100

Sample 8- Beta cateninIP Mild KC 100-25

Sample 9-Beta catenin Ip Mild 25 below

Sample 1

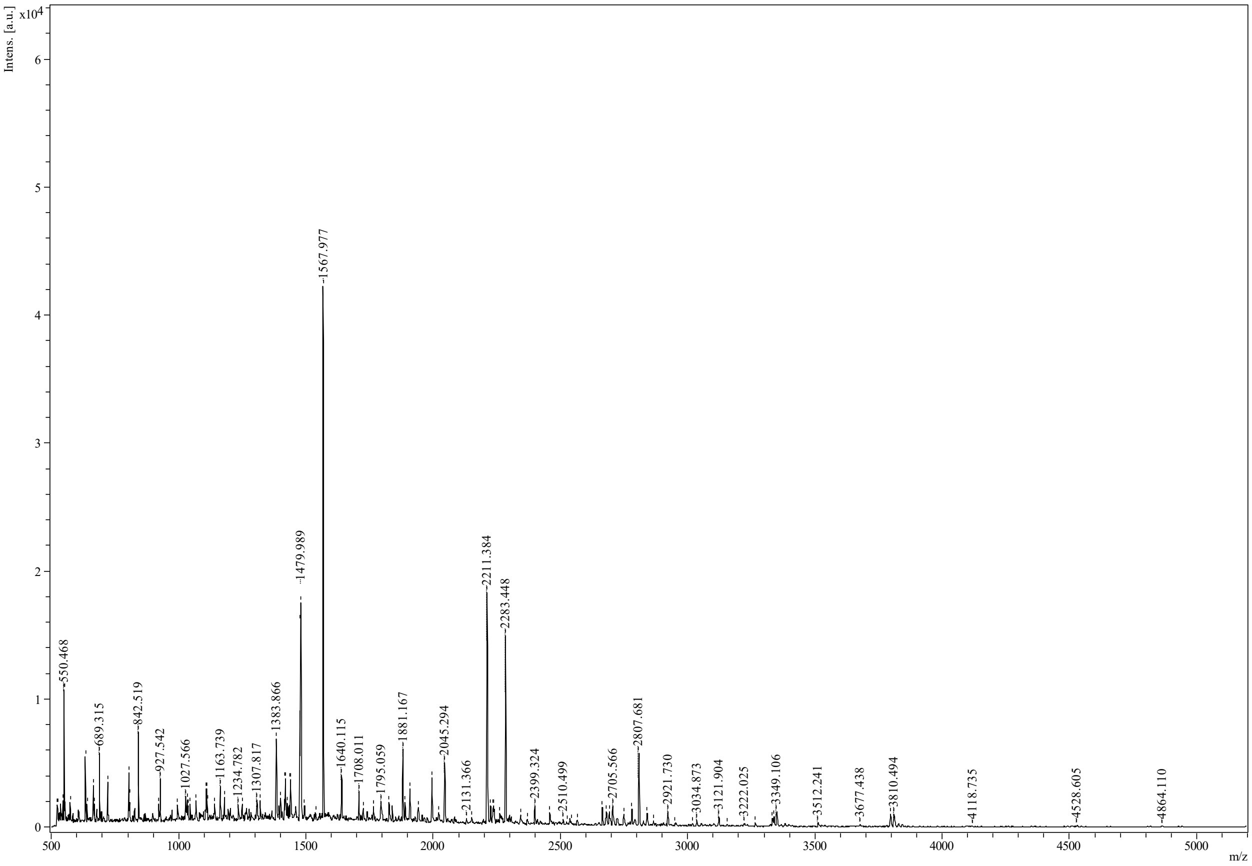

m/z	S/N	Quality Fac.	Res.	Intens.	Area
522.512	9	509	555	1625	2180
524.321	6	380	1992	1168	438
544.861	9	988	3064	1675	484
550.468	56	20026	3516	10314	2650
553.253	8	2770	2183	1383	543
573.947	9	1037	2728	1583	549
634.172	29	6558	3977	5248	1455
640.498	8	1755	3825	1424	422
665.304	15	9341	3797	2850	828
668.547	8	3089	4033	1447	433
689.315	29	31887	4222	5364	1507

721.303	17	12338	4153	3197	992
804.262	21	11080	4236	4095	1509
807.394	8	3487	4197	1516	530
842.519	37	109684	4691	7053	2380
922.527	8	1467	5226	1518	529
927.542	18	41809	5022	3602	1298
995.585	7	956	5330	1485	560
1027.566	10	2204	5265	2077	855
1033.600	9	4318	5351	1793	745
1036.599	6	1152	5285	1297	566
1045.637	7	4874	5383	1449	591
1068.580	9	4102	5204	1866	830
1106.657	6	667	4366	1294	749
1109.618	12	16082	4909	2594	1309
1112.659	7	3651	6208	1525	596
1141.632	7	1319	3062	1464	1268
1163.739	14	4410	5154	2956	1501
1179.721	9	4023	5752	2068	957
1234.782	7	1946	4825	1478	926
1249.754	6	2196	5377	1364	793
1307.817	7	2266	5061	1646	1087
1320.759	7	3517	5607	1638	964
1383.866	33	21802	5269	7332	4898
1399.869	10	7217	5373	2151	1480
1415.865	9	3995	5448	1948	1340
1419.880	16	10405	5184	3538	2594
1427.894	7	6154	5875	1449	927

1434.914	7	2834	4581	1409	1219
1439.973	15	9478	5500	3310	2238
1475.944	43	7603	5750	9229	6288
1479.989	87	31687	5425	18722	13581
1493.948	4	5485	1647	513	
1537.972	3	4591	1156	414	
1567.977	206	1.000623e6	4398	42411	43507
1640.115	17	38115	4922	3404	3335
1708.011	13	29407	5530	2413	2286
1725.073	4	5758	1460	488	
1765.992	3	4828	1360	539	
1795.059	8	3312	4581	1529	2050
1827.045	8	10959	4601	1441	1957
1881.167	31	69077	5574	5269	5904
1889.156	8	10369	5049	1387	1820
1908.158	15	12809	5321	2461	3003
1941.156	7	2364	4418	981	1601
1994.233	21	27025	5291	3077	3954
2020.246	2	5159	872	373	
2045.294	29	58847	5453	3967	4945
2131.366	2	5518	685	282	
2150.325	2	5468	595	261	
2211.384	132	57948	5670	14304	19299
2225.385	10	28871	5502	1039	1483
2235.386	10	3961	4167	989	2060
2239.403	7	14527	4838	739	1237
2262.436	6	1014	3752	559	1293

2283.448	102	47127	5463	10238	15494
2285.452	31	2199	5950	3188	4043
2343.351	3	5645	893	424	
2369.425	2	4324	553	328	
2399.324	12	26574	4590	1086	1929
2458.490	8	9547	5356	668	1108
2510.499	2	6044	555	248	
2525.525	2	5264	402	188	
2542.534	2	6572	452	191	
2567.560	2	5565	511	263	
2663.584	17	3044	4864	976	2194
2680.613	11	7403	4848	665	1373
2691.617	12	1214	4505	675	1673
2705.566	18	13282	4820	1075	2239
2748.653	11	2313	4938	566	1321
2780.453	12	3935	2609	638	2439
2807.681	69	14082	5140	3580	8113
2839.638	13	2456	4719	614	1585
2865.714	2	6326	407	213	
2921.730	14	4964	4810	607	1616
2950.765	2	6585	248	122	
3019.772	2	5660	257	154	
3034.873	6	2387	4666	260	718
3121.904	11	6686	4928	395	1127
3156.963	1	5620	167	109	
3222.025	2	7241	253	144	
3265.981	2	6109	235	151	

3332.054	12	2576	4831	324	1089
3338.241	13	4912	4403	330	1202
3349.106	21	3553	5013	541	1763
3512.241	7	2892	5032	150	503
3677.438	3	7827	176	115	
3795.642	15	1003	1958	174	1578
3810.494	20	3130	4760	389	1320
4118.735	2	6091	91.5	71.8	
4528.605	2	12041	66.0	31.7	
4864.110	2	7687	69.5	52.4	

Sample 2

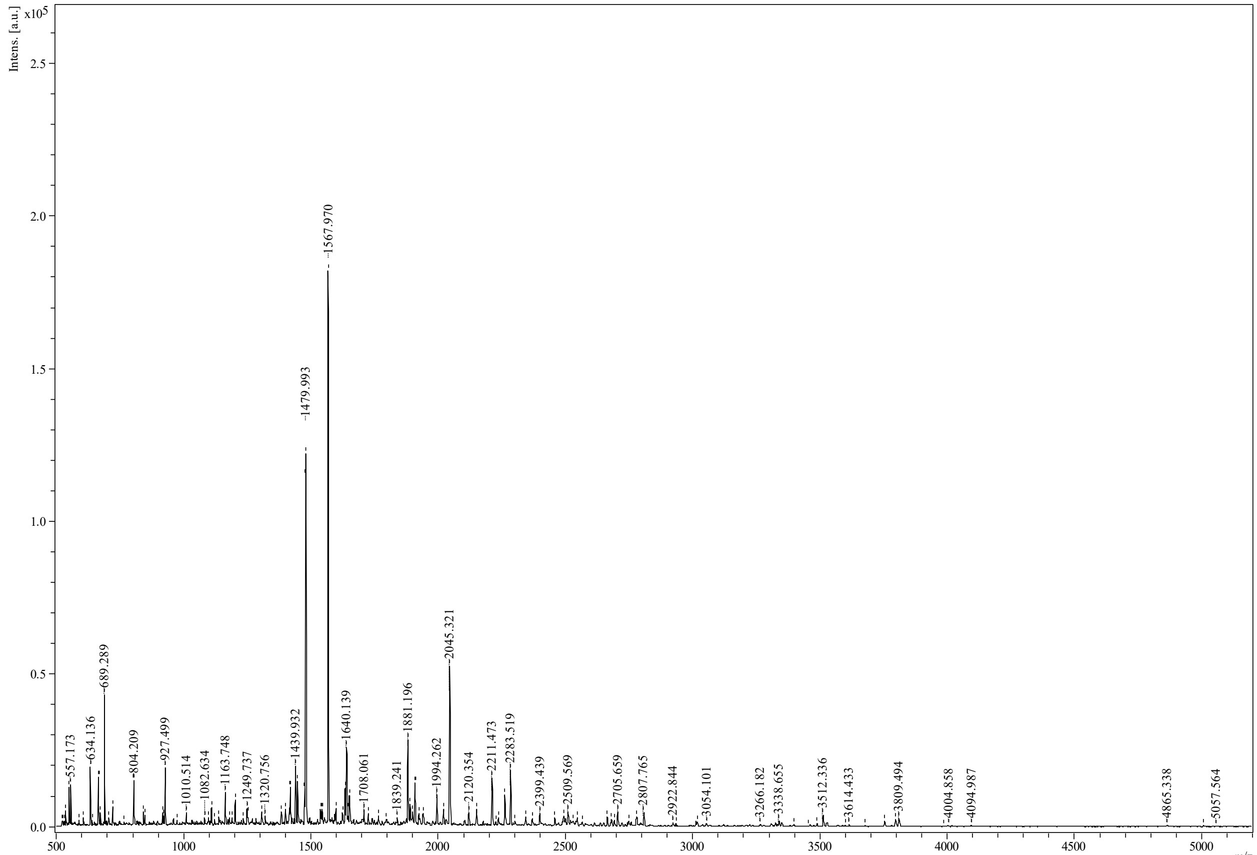

m/z	S/N	Quality Fac.	Res.	Intens.	Area
522.901	8	678	424	1712	3185

524.325	6	180	2652	1424	401
531.156	7	340	1604	1683	796
537.124	22	5540	2398	5160	1938
550.457	57	18346	3389	13187	3467
557.173	59	91272	3013	13804	4203
589.160	7	1856	2808	1733	629
606.099	11	7941	3076	2487	871
634.136	83	23734	3664	19430	5899
640.481	7	7917	4173	1761	444
665.295	66	196185	3263	15728	5635
668.521	7	5461	4096	1715	494
673.285	18	32664	3277	4301	1584
689.289	176	312236	3335	42222	15785
706.307	8	9240	3564	1910	685
721.287	26	18294	3352	6230	2472
765.343	6	1904	3238	1474	667
802.399	6	770	2680	1524	875
804.209	60	50660	4065	14931	5845
842.475	17	50359	4622	4280	1459
847.497	13	8699	3586	3381	1520
917.541	16	39534	3402	4222	2344
922.507	12	50361	3214	3024	1796
927.499	72	271141	3935	18979	9120
973.533	7	3269	3393	1937	1214
1010.514	14	50592	3658	4056	2444
1082.634	9	19581	3705	2574	1716
1098.650	8	18603	2760	2377	2249

1109.624	17	29150	3248	5322	4207
1121.591	6	10778	3075	1857	1564
1138.582	9	4322	3313	2971	2490
1163.748	34	63375	3314	10685	8907
1179.669	8	5086	3479	2672	2267
1189.683	6	5420	2705	2013	2300
1202.711	26	37578	3320	8593	7819
1234.736	7	7462	3148	2165	2183
1249.737	17	175026	3440	5588	5083
1252.742	7	7832	3157	2205	2261
1305.841	13	9290	2879	4705	5706
1320.756	8	16642	3580	2830	2649
1383.859	13	52517	3346	4629	5139
1399.881	14	213962	3553	5028	5244
1415.888	11	10833	2956	4145	5563
1417.931	7	1461	6141	2512	1667
1419.899	30	383470	3236	11029	12849
1439.932	50	480837	3751	18931	20215
1447.901	40	38779	3811	14986	16087
1473.914	8	1243	3572	2955	3688
1475.955	28	3188	3059	10667	14431
1479.993	346	174805	3362	131850	163273
1538.006	12	33978	3462	4673	6013
1541.982	6	3085	3358	2376	3119
1544.956	7	1822	1839	2649	5748
1567.970	473	1.098196e6	3467	185165	244517
1597.999	12	82028	3403	4532	6392

1625.032	9	46632	3265	3359	4896
1635.122	30	25790	3297	11870	18108
1637.988	9	1209	4737	3743	4390
1640.139	62	368632	3274	24589	35922
1646.024	7	7763	2974	2641	4630
1652.023	25	119531	3521	9996	14407
1708.061	9	35994	2905	3714	6909
1725.084	9	103311	3244	3495	5668
1766.043	6	12115	3073	2376	4435
1795.142	2	4162	2064	1078	
1839.241	3	4007	2929	1487	
1881.196	70	152976	3361	25319	47629
1889.187	18	46895	3185	6020	12557
1899.253	10	28396	3306	3577	6922
1908.199	33	39866	3139	10447	23359
1911.223	8	12839	3077	2614	5896
1924.177	10	2862	3392	3032	6448
1941.239	9	7379	3059	2784	6210
1994.262	25	104075	3702	8291	15325
2020.322	13	9041	2899	3838	9824
2045.321	140	168720	3500	43443	88458
2120.354	11	18564	3377	3327	6661
2150.396	15	73820	3443	4287	8942
2211.473	40	72281	3304	10616	25447
2226.510	2	4655	1558	884	
2236.548	4	4388	3067	1970	
2260.469	8	338	5231	2322	3269

2283.519	52		17462		3413		12304		31716
2301.494	2		4479		1560		900		
2343.422	8	8304		3458		2103		4703	
2369.429	7	2414		3299		1547		4174	
2399.439	13		15766		3578		2906		6648
2458.554	9	10240		3669		1921		4380	
2492.551	12		3355		3563		2164		6101
2509.569	14		1699		2950		2418		8141
2529.184	6	181		5074		1306		2269	
2545.617	4		4566		2346		1586		
2565.599	3		4796		1372		853		
2663.638	12		7047		3349		1865		6077
2680.638	9	3701		3501		1470		3955	
2691.672	8	4403		3390		1238		3925	
2705.659	18		30299		3807		3075		7853
2748.720	7	5424		3297		1063		3512	
2779.471	8	1105		1404		999		7250	
2807.765	19		15697		3072		2394		9153
2922.844	3		5601		1090		743		
2936.884	1		5550		495		337		
3018.013	6	6192		3940		724		2153	
3054.101	3		5954		1030		880		
3266.182	3		7898		759		623		
3338.655	8	2165		1854		510		3842	
3396.452	2		6297		461		361		
3455.394	1		5596		231		167		
3488.368	3		5548		687		602		

3512.336	26	12128	3054	1316	6945
3599.459	3	6255	485	410	
3614.433	3	7601	502	407	
3678.524	2	5146	322	309	
3753.602	17	34216	3961	709	2937
3795.880	12	5303	1585	421	4419
3809.494	25	20443	2965	738	4896
3987.760	2	6287	244	231	
4004.858	3	11442	335	238	
4094.987	3	6084	349	328	
4865.338	6	7053	411	498	
5006.679	3	10435	211	188	
5057.564	5	2742	303	706	

Sample 3

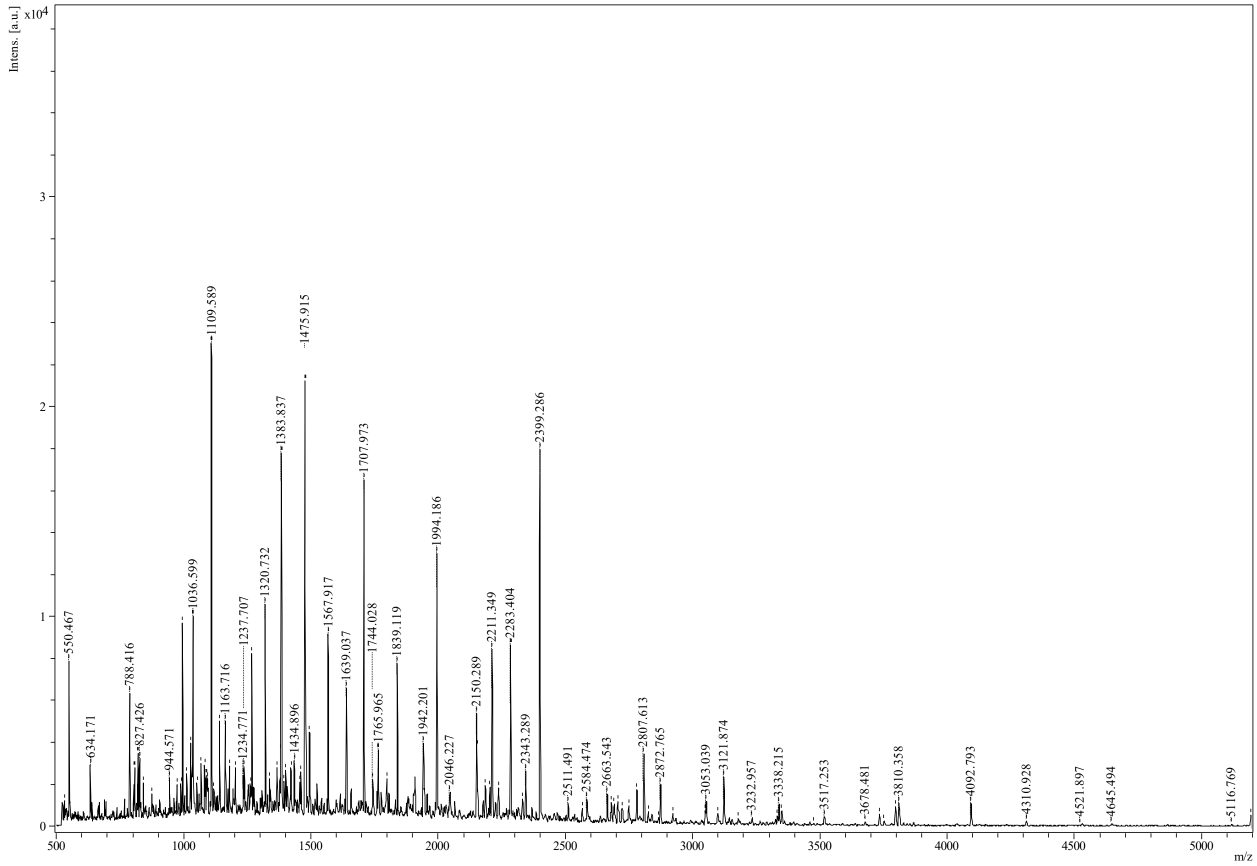

m/z	S/N	Quality Fac.	Res.	Intens.	Area
531.223	7	291	1938	1157	453
550.467	46	9982	3661	7749	1865
634.171	15	7060	3384	2684	903
788.416	33	24248	3927	6205	2365
804.259	9	5880	2959	1719	966
807.393	13	4276	4025	2470	946
818.383	10	1915	3988	1881	758
820.394	16	15187	4045	3033	1194
822.419	6	3640	4611	1268	434
827.426	16	7905	4212	3066	1162
842.501	9	4166	4398	1693	625

874.462	7	707	3919	1409	627
944.571	9	4323	4292	1976	903
973.549	7	3293	4030	1548	813
989.616	7	2807	3854	1516	844
995.575	42	76951	4874	9355	3978
1012.527	10	7309	4611	2184	1041
1027.558	15	11802	4980	3533	1546
1033.586	11	2098	4139	2567	1437
1036.599	43	39703	4938	9863	4418
1052.593	7	3046	4737	1543	761
1068.578	12	2608	4966	2824	1348
1082.660	12	4127	4656	2739	1433
1086.598	9	4051	4893	2169	1088
1090.615	9	2316	4747	2223	1161
1093.591	7	257	5636	1684	765
1107.629	10	1123	4370	2343	1414
1109.589	93	98487	4969	22528	11206
1118.607	6	958	4477	1547	918
1141.568	18	16309	4671	4478	2567
1163.716	19	13839	4691	4681	2688
1179.703	12	2723	4685	2910	1761
1203.722	9	4178	4431	2289	1540
1234.771	7	4966	3853	1829	1486
1237.707	9	6141	5001	2289	1407
1262.721	7	1333	4392	1762	1307
1267.814	29	26254	4740	7226	4878
1320.732	38	73557	5317	9734	6081

1337.823	7	1236	4443	1708	1374
1365.789	7	2806	4822	1728	1333
1367.802	7	4063	4929	1836	1383
1378.800	7	267	5503	1707	1147
1380.792	8	181	5122	1912	1450
1383.837	64	19765	5157	16310	11463
1385.879	7	466	6070	1875	1136
1393.840	6	2180	4635	1580	1325
1400.834	8	7441	5195	2076	1525
1421.839	10	8317	5080	2465	1898
1434.896	10	18102	4620	2540	2206
1455.843	7	4090	5075	1660	1364
1460.927	8	9013	3911	2069	2219
1475.915	91	40659	5008	22641	18577
1479.950	20	20383	4812	4910	4261
1493.892	18	18773	5038	4519	3811
1567.917	37	268444	5414	8878	7431
1639.037	26	33166	4882	6034	6263
1707.973	69	373764	5066	15903	16708
1744.028	7	5585	4375	1604	2191
1763.996	7	5603	5234	1475	1626
1765.965	11	92191	4900	2379	2768
1798.117	8	18931	3982	1693	2613
1839.119	33	178277	5192	7085	8328
1942.201	17	107702	4852	3272	4541
1994.186	62	157054	5124	11203	14985
2046.227	6	3201	3569	948	2093

2150.289	29	167558	4977	4185	6346
2155.305	8	30038	4723	1113	1802
2185.356	10	5177	4386	1314	2444
2202.357	8	13278	4343	1132	2128
2211.349	46	64397	4802	6415	10547
2236.374	10	1955	3792	1194	2735
2283.404	47	11708	4307	5693	11651
2286.405	9	802	5519	1149	1567
2331.392	6	8711	4337	738	1454
2343.289	15	28798	4426	1788	3207
2399.286	119	233324	4879	13553	22863
2511.491	3	5371	954	486	
2565.492	2	4913	561	310	
2584.474	8	4998	4514	707	1602
2663.543	12	4640	4382	894	2231
2679.485	8	1436	4746	582	1398
2691.570	8	2199	4362	590	1480
2705.483	9	776	4253	620	1657
2721.527	8	993	4479	537	1384
2748.642	7	3571	4320	515	1309
2781.045	11	571	2972	819	2749
2807.613	30	4710	4544	1990	5259
2824.760	2	5722	406	203	
2865.687	2	5130	365	195	
2872.765	17	17143	4905	1110	2677
2921.771	2	5368	428	240	
3048.916	7	3372	4887	375	926

3053.039	11	7843	4409	599	1706
3097.918	2	5864	380	221	
3121.874	25	7610	4455	1120	3597
3177.918	1	5401	224	146	
3232.957	2	6809	292	193	
3332.001	7	1852	3998	201	815
3338.215	13	5290	3904	386	1596
3349.086	10	4625	4215	327	1194
3475.041	1	9898	111	48.3	
3517.253	7	1327	4453	215	713
3678.481	3	5780	213	168	
3732.302	11	6091	4339	202	860
3751.407	1	8700	81.3	45.0	
3795.419	18	6091	3913	325	1544
3810.358	17	2564	4032	387	1549
4092.793	27	6846	5059	382	1571
4310.928	4	5981	175	161	
4521.897	1	18451	41.9	18.5	
4645.494	1	11254	54.9	39.8	
5116.769	1	12028	43.3	23.2	
5191.688	12	4645	4198	86.8	547

Sample 4

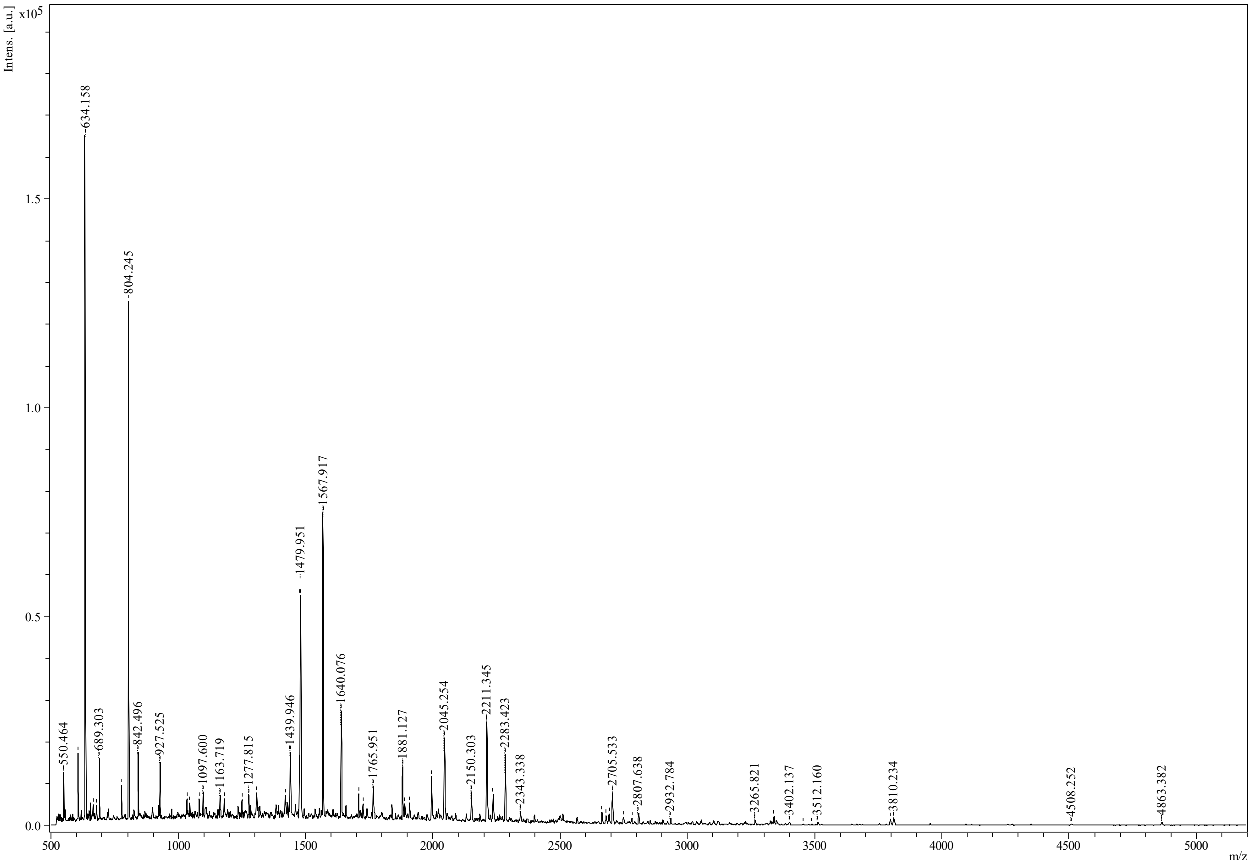

m/z	S/N	Quality Fac.	Res.	Intens.	Area
550.464	22	7250	3433	12011	3157
606.112	30	9549	3431	16877	5122
634.158	293	73799	3427	164108	52706
636.186	12	2012	3227	6750	2338
665.283	7	1797	3208	3684	1367
678.247	7	2725	3520	3914	1367
689.303	27	10936	3588	15218	5171
776.196	15	8898	3416	8620	3886
804.245	221	62737	3774	126663	51814
806.272	26	15581	3315	14628	7044
842.496	28	51606	3761	16056	6804

927.525	23	63212	4227	14094	6230
1033.578	8	3524	4273	4890	2626
1045.607	6	3644	4096	3869	2206
1082.641	8	7129	4002	5179	3157
1097.600	10	19397	4421	6657	3762
1163.719	10	3658	4370	6152	3862
1179.695	8	3583	4629	5279	3214
1249.740	8	5532	4756	5132	3384
1277.815	9	17855	4538	5589	3974
1305.823	7	4419	4629	4739	3453
1307.796	8	3320	4158	5377	4427
1419.842	9	5271	4361	6065	5507
1435.853	6	5299	4122	3932	3852
1439.946	24	72451	4470	15665	13700
1475.905	25	9294	4313	16003	15666
1479.951	92	77988	4469	58177	53929
1567.917	118	852677	4403	73063	75422
1638.000	14	363	9721	8683	4538
1640.076	39	216752	4333	23289	26421
1707.984	11	30054	4322	6199	7942
1725.024	6	10148	4431	3563	4623
1765.951	14	45485	4232	7929	11014
1881.127	24	210830	4460	12075	17647
1889.141	7	16318	4188	3650	5812
1908.123	7	8778	3989	3493	6148
1994.187	21	34598	4330	8999	14968
2045.254	41	119064	4354	16957	28288

2150.303	16		91661		4074		5485		10554
2211.345	56		81213		4268		18700		35690
2235.377	16		2882		3568		4767		11763
2283.423	40		9433		3836		11647		26776
2343.338	7	17656		3745		2048		4506	
2663.567	10		5949		3963		1814		5053
2680.596	7	4443		3817		1335		3324	
2691.595	9	2441		3774		1726		4334	
2705.533	29		170008		4161		5167		13044
2748.664	6	2656		3636		1011		3188	
2782.637	2		5377		1445		1002		
2807.638	12		6141		4144		1765		5135
2932.784	6	7358		4093		827		2674	
3265.821	6	1346		3400		567		2196	
3338.346	9	1676		1849		497		3913	
3402.137	3		5914		754		583		
3454.057	1		8418		257		158		
3489.272	1		4917		310		252		
3512.160	2		8218		364		250		
3795.710	12		2779		2292		364		2742
3810.234	13		1707		3031		538		2870
4508.252	7	175		8666		137		407	
4863.382	9	12367		3830		151		949	

Sample 5

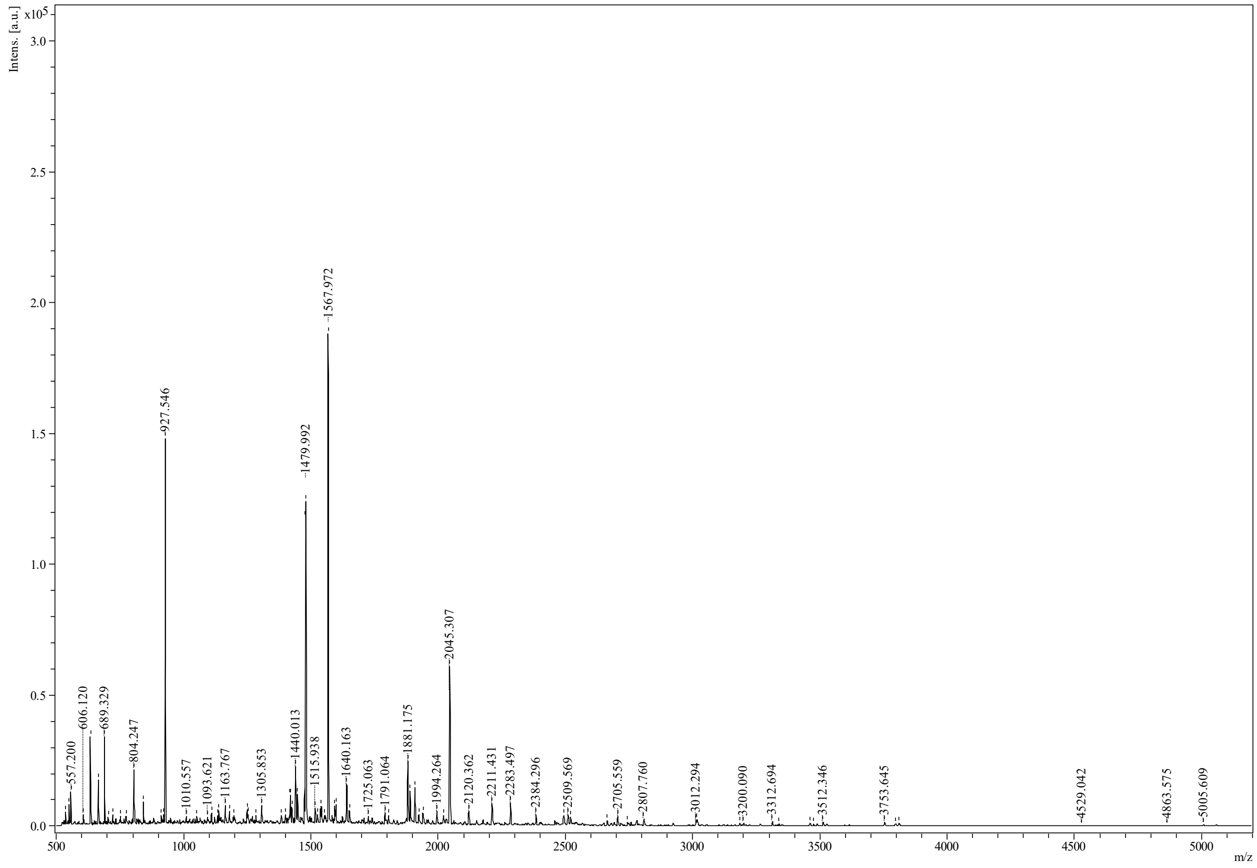

m/z	S/N	Quality Fac.	Res.	Intens.	Area
537.148	13	3869	2398	4579	1737
550.467	21	7017	3736	7596	1824
557.200	34	160264	2809	12306	4068
606.120	11	8671	2712	3819	1548
634.162	92	122230	2828	33168	13741
665.333	47	114170	3339	16921	5903
689.329	91	633788	2978	33178	14026
704.345	7	2643	2932	2747	1243
721.316	10	5235	3242	3875	1607
752.322	8	4195	3417	3106	1334
774.363	8	1210	2717	2856	1620

804.247	56	50518	2898	20819	11911
806.451	19	19212	2271	7092	4896
842.521	21	46314	3343	8043	4073
912.512	8	3959	3414	3324	1874
922.538	9	6578	3346	3526	2049
927.546	375	472442	3343	148746	87031
1010.557	7	8952	3828	3054	1782
1051.549	7	1179	3646	2954	1965
1093.621	7	1651	3558	2981	2169
1109.651	8	4481	3392	3644	2804
1132.646	7	7004	4180	3023	1944
1138.652	12	13640	3730	5372	3949
1163.767	16	11669	4007	7206	4940
1179.712	11	3844	3686	4798	3829
1198.804	7	1388	3138	2909	2783
1249.770	12	69455	4215	5651	4235
1252.759	7	1745	4164	3262	2498
1283.837	6	4499	3697	2750	2523
1305.853	16	10826	3797	7272	6642
1383.868	8	6860	3939	3578	3462
1399.887	8	31317	4453	3586	3027
1415.905	6	1026	4237	2769	2545
1417.946	8	591	6738	3753	2089
1419.919	21	231155	4091	9673	8916
1425.901	14	51049	3976	6636	6509
1440.013	47	342362	4174	21573	20358
1447.973	27	18326	4575	12458	10805

1474.957	22	11410	3380	10117	13366
1479.992	287	238365	3875	131841	144553
1515.938	13	10709	3658	6051	7599
1525.961	7	4490	3506	3082	4105
1537.998	12	9978	4173	5514	5979
1540.950	13	3337	3456	5850	8149
1552.885	7	1627	3193	3018	4742
1567.972	419	1.408708e6	3762	191357	235136
1594.060	13	43239	3851	5942	7400
1598.006	15	98345	4152	6676	7890
1640.163	33	431370	3847	14744	19502
1652.034	11	67579	3802	4814	6696
1725.063	7	55124	3905	2899	4355
1791.064	10	2434	3344	3717	7225
1805.156	7	7170	3313	2622	5191
1881.175	59	415668	3993	21971	37202
1889.182	31	97863	3992	11526	19732
1908.217	34	35258	3982	12216	21321
1924.222	9	8092	3541	2919	5970
1940.227	11	5907	3594	3744	7629
1994.264	13	184742	4111	4407	7576
2020.278	9	3323	3499	2721	5945
2045.307	160	485859	4034	50628	93141
2120.362	14	192683	4016	4303	7676
2211.431	24	47474	3883	6066	13003
2283.497	26	20054	3873	6025	13851
2384.296	12	5741	3551	2703	6032

2492.617	14	7400	3616	2413	6689
2509.569	16	4156	3266	2635	8027
2519.662	12	8961	3322	1914	5826
2663.654	8	16658	3730	1112	3271
2705.559	15	26058	3907	2332	5784
2743.729	6	656	4333	853	2323
2807.760	13	17358	3768	1585	5054
3012.294	9	1070	2830	834	3603
3017.102	11	1783	2332	832	4465
3184.074	6	10794	3353	466	1841
3200.090	6	15602	3519	501	1875
3312.694	12	14445	4541	803	2464
3338.610	6	706	5072	333	1127
3460.176	8	3839	3188	417	1921
3474.577	6	417	7000	316	823
3512.346	12	10200	3247	522	2518
3753.645	14	32367	3984	514	2080
3795.596	8	8047	1705	181	1876
3809.625	11	20302	2492	253	1936
4529.042	6	151	8088	90.1	291
4863.575	6	161	7800	68.5	256
5005.609	7	2926	3023	51.5	475

Sample 6

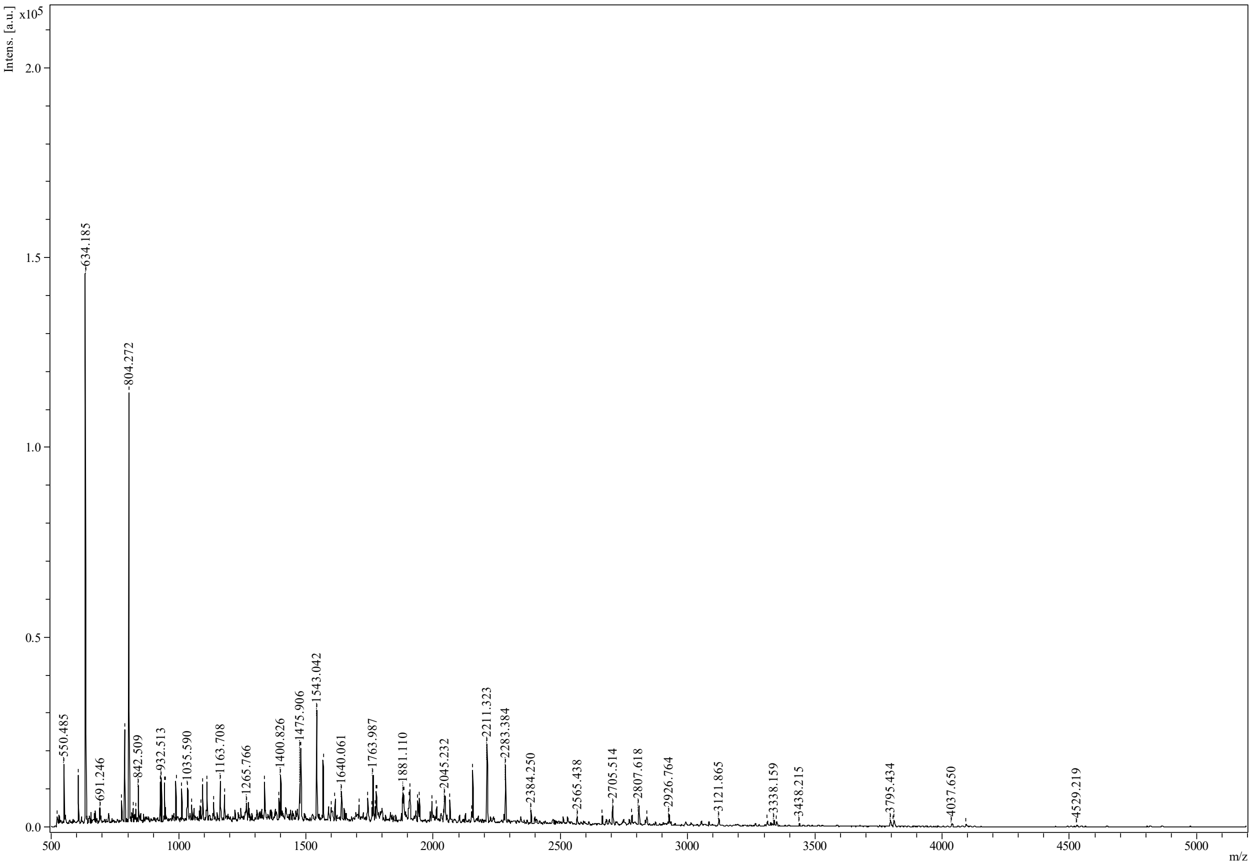

m/z	S/N	Quality Fac.	Res.	Intens.	Area
522.654	6	149	800	3164	2938
550.485	31	14529	2841	15836	5111
606.136	25	18186	2774	13022	5059
634.185	273	176791	2857	144634	56980
636.214	14	2460	3020	7301	2705
691.246	7	2072	3293	3997	1553
776.225	11	11182	2942	6241	3308
788.422	45	156213	3085	25187	12128
804.272	207	68251	3319	115647	54695
806.312	28	22498	2973	15522	8219
820.385	7	6114	2663	4175	2548

831.469	6	5326		3040		3496		1909	
842.509	17		14684		3722		9532		4179
927.531	18		35878		4248		10822		4773
932.513	20		64688		4006		11961		5697
944.561	18		29607		4077		10891		5215
948.514	7	4873		3907		4415		2233	
989.615	18		25172		4061		10984		5743
1012.523	15		28359		4219		9150		4715
1032.623	7	1178		3910		4311		2569	
1035.590	17		5253		4036		10533		6016
1051.580	8	4720		3863		4868		3012	
1086.583	8	5892		4097		5147		3137	
1094.618	16		12891		4403		10457		5874
1111.641	16		28148		4510		10731		6026
1137.652	8	5603		4215		5356		3361	
1163.708	17		12553		4355		11491		7279
1180.715	12		6135		4639		8299		5086
1265.766	6	2100		3346		4170		3938	
1337.847	15		14317		4463		10395		8171
1394.875	9	20348		4940		6144		4673	
1400.826	18		55507		5209		12606		9117
1475.906	16		28930		4786		10849		9451
1479.945	30		69303		4910		20639		17508
1543.042	41		177914		4897		28375		25891
1567.925	24		224633		4680		16248		16352
1598.922	6	3004		4568		4297		4783	
1613.993	6	1642		3340		4111		6548	

1640.061	10	49644	4965	6959	7034
1707.948	7	11216	4378	4324	5806
1743.999	10	46544	5046	6120	7207
1759.995	6	6569	4702	3840	5063
1763.987	20	29015	4719	12504	16102
1772.181	6	26832	4116	3808	5557
1776.011	8	4350	5208	4888	5474
1778.033	10	6123	4722	6218	8069
1881.110	12	67054	5114	6678	8991
1884.130	10	23296	5314	5681	7261
1906.184	7	261	10122	4053	2584
1908.125	11	5630	4408	6039	9725
1940.137	9	8231	4403	4552	7582
1946.190	10	46410	4606	5002	7857
1994.168	10	42786	5722	4994	6274
2013.177	7	16206	4439	3244	5572
2045.232	13	5216	4482	5606	9640
2065.238	11	31058	4513	4741	7969
2150.267	6	19453	5324	2417	3641
2155.291	29	541730	5493	11552	16076
2211.323	44	245206	5623	16757	23735
2283.384	33	26732	5149	11446	19360
2384.250	9	72168	4825	2824	5065
2565.438	6	31827	4584	1429	3078
2663.549	8	23434	5341	1588	3253
2705.514	16	132189	5291	3216	6318
2780.366	6	1887	1912	1000	5228

2807.618	19	4807	5072	3082	7360
2838.741	7	7720	4062	1045	3157
2926.764	11	30103	4951	1635	4101
3121.865	10	6498	5102	1062	2988
3312.786	7	2061	3933	627	2156
3338.159	10	2685	5206	664	2102
3349.072	7	2992	4443	437	1594
3438.215	1	7658	366	183	
3795.434	18	4429	5718	635	2126
3808.472	6	538	10466	267	422
3810.495	14	5455	5225	577	1789
4037.650	11	31921	6413	312	952
4093.835	8	2864	5699	226	771
4529.219	6	193	5569	113	431

Sample 7

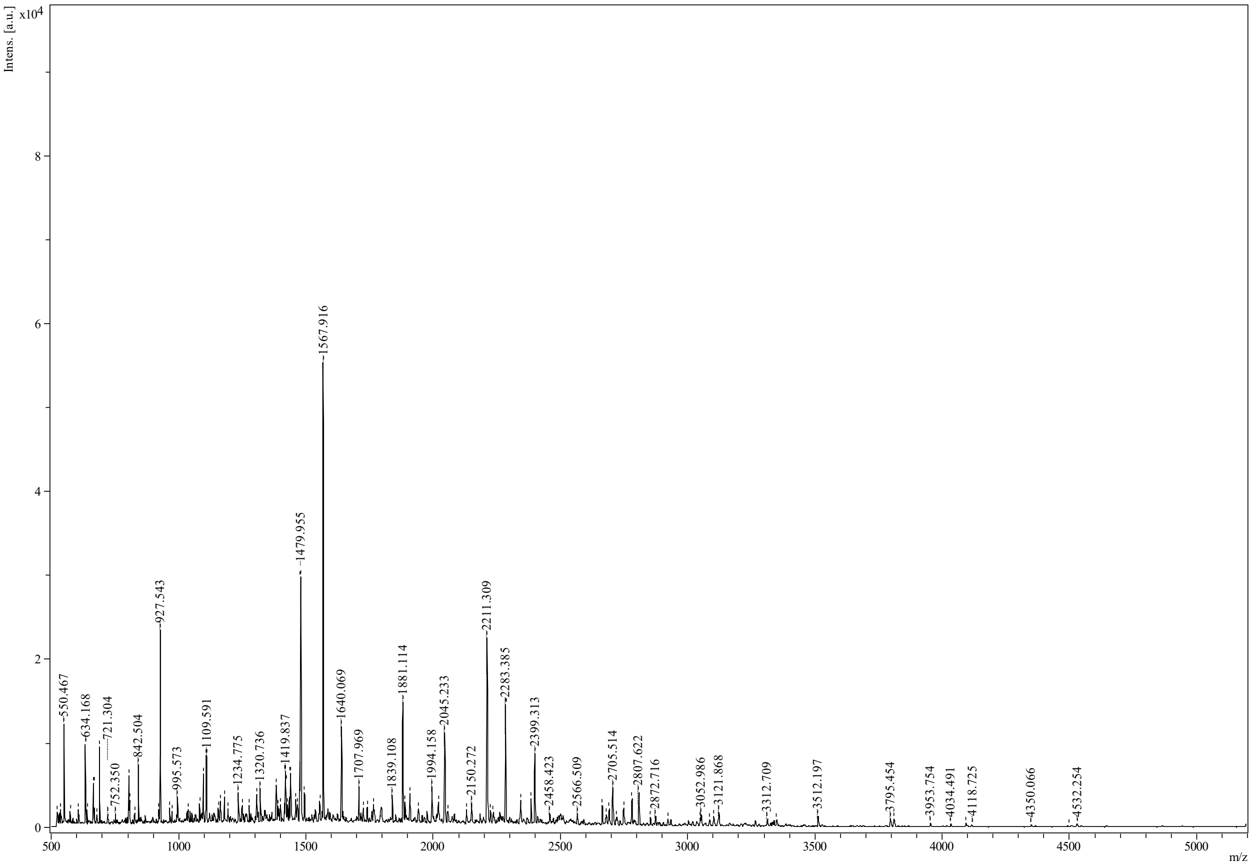

m/z	S/N	Quality Fac.	Res.	Intens.	Area
522.589	11	163	706	1996	2102
537.163	7	382	1297	1244	815
550.467	64	10470	3558	12056	3041
573.937	9	906	3289	1658	505
606.119	9	1514	3268	1745	576
634.168	51	14024	3424	9789	3267
640.505	9	3980	4149	1793	466
665.300	25	13568	4129	4886	1352
668.541	10	2856	3602	1878	635
678.254	6	778	3555	1215	425
689.307	47	37097	4226	9189	2662

721.304	7	766	3898	1298	450
752.350	6	3866	3344	1300	561
804.253	29	16227	3697	5836	2560
807.382	13	8590	4315	2703	962
827.430	7	839	4657	1422	476
842.504	34	31585	5036	7036	2242
922.513	8	1268	5193	1764	632
927.543	105	642017	3876	23104	11561
965.498	9	5376	4237	2016	996
973.556	7	1505	4944	1575	655
995.573	14	7903	5218	3149	1245
1036.588	7	583	5449	1715	730
1082.655	10	5241	4960	2466	1200
1097.600	23	48166	4721	5833	3146
1107.626	11	5913	4520	2752	1604
1109.591	30	44871	5517	7547	3383
1156.643	8	2751	5597	2107	1022
1163.705	10	6455	5111	2687	1443
1179.699	12	9152	5601	3172	1577
1193.701	7	927	5070	1751	1018
1234.775	14	5673	5192	3868	2249
1249.735	9	2682	5878	2563	1347
1277.806	8	5517	6114	2218	1151
1305.825	13	7726	5204	3751	2423
1320.736	15	8822	5395	4116	2573
1383.830	17	17275	6320	4890	2757
1389.812	7	1059	5665	2053	1389

1399.816	7	2439	6238	2060	1285
1415.834	9	5585	6343	2562	1514
1419.837	22	44838	6166	6224	3787
1421.881	9	5828	5813	2569	1706
1427.863	7	8395	5597	2129	1505
1434.882	11	5397	5332	3202	2398
1439.932	21	19980	6088	6019	3842
1460.901	7	3840	5304	2101	1618
1466.828	8	6173	4603	2373	2206
1475.906	28	13592	5564	7933	5891
1479.955	107	131827	5511	30714	23010
1493.885	13	16712	5772	3782	2808
1554.912	9	15913	5707	2435	1943
1567.916	190	245257	5938	54221	41342
1638.001	11	183	11278	3008	1317
1640.069	37	83798	5527	10333	9217
1707.969	15	42722	5773	4160	3962
1724.996	6	4638	5818	1712	1710
1740.977	7	14556	5715	2006	2051
1765.961	9	4534	5425	2428	2628
1839.108	11	30241	6034	3029	3190
1881.114	54	301439	6137	13994	14805
1889.102	9	11758	5439	2338	2934
1908.096	13	15040	5758	3313	3922
1942.141	7	16323	6000	1566	1799
1994.158	17	9177	5527	3835	4918
2020.074	9	1511	5096	1826	2858

2045.233	42	202429	5797	9203	11446
2057.190	6	4360	5315	1317	1896
2131.307	7	20422	5934	1437	1825
2150.272	10	52955	5691	2026	2765
2211.309	97	270083	5980	17798	23643
2225.311	7	51596	5945	1267	1742
2235.316	6	881	4545	1042	2051
2283.385	63	45089	5589	10618	16272
2286.370	8	927	5786	1416	1903
2343.289	13	53946	5322	2111	3362
2384.181	15	74191	5904	2229	3397
2399.313	41	210717	5804	6099	8992
2458.423	7	14563	5896	948	1454
2566.509	6	3518	5343	786	1388
2663.552	16	16810	5634	1641	3105
2679.532	7	2766	4618	702	1729
2690.554	9	1208	3675	874	2657
2705.514	30	179090	5629	3044	5653
2720.552	7	1582	4655	642	1570
2748.685	13	3035	4693	1261	2632
2780.417	17	2819	2777	1591	5730
2807.622	27	4709	4975	2304	5610
2853.725	6	5458	5546	561	1125
2872.716	8	12119	5489	680	1499
2921.714	6	6737	4424	454	1302
3048.868	6	2510	4238	455	1251
3052.986	9	5572	4131	643	1875

3085.820	6	8374	5701	440	963
3101.803	10	3757	5491	649	1610
3121.868	14	11577	5611	903	2217
3312.709	9	3180	4659	433	1257
3349.055	9	949	5198	356	1119
3512.197	16	27779	5851	603	1668
3795.454	15	5866	5383	346	1221
3810.486	11	4075	4911	305	1005
3953.754	4	8662	355	235	
4034.491	4	7656	345	235	
4093.840	4	7213	316	234	
4118.725	3	7608	205	141	
4350.066	2	9749	127	79.0	
4498.190	2	18237	89.2	50.3	
4532.254	6	9278	328	238	

Sample 8

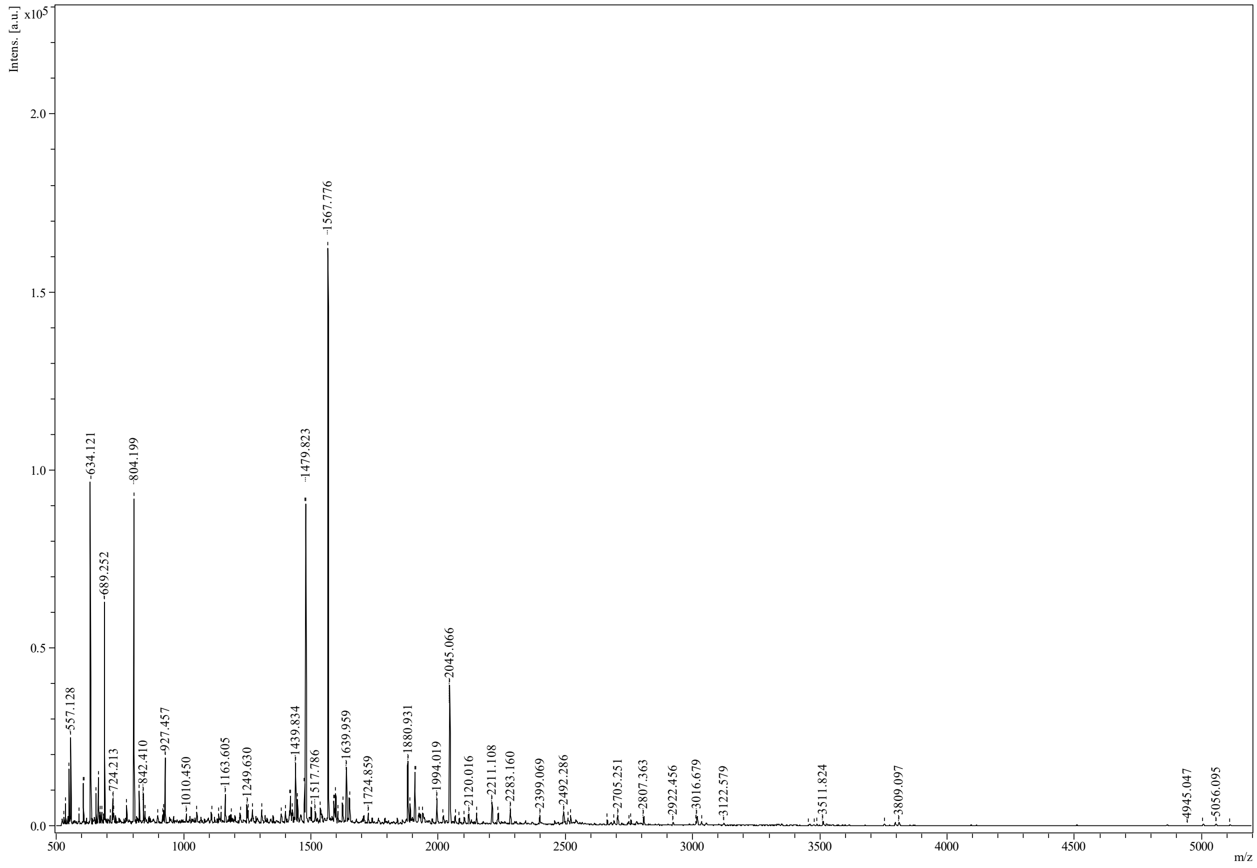

m/z	S/N	Quality Fac.	Res.	Intens.	Area
530.011	6	457	2661	2581	835
537.084	15	4862	3441	6472	1593
550.429	39	10959	4125	16584	3385
554.119	7	397	3126	2936	824
557.128	60	41115	3944	25182	5550
589.109	8	1817	4020	3168	753
606.079	29	12034	4347	12124	2740
609.135	6	2704	4286	2564	579
634.121	234	40381	4425	98215	23318
636.148	8	547	4698	3491	773
656.113	20	15349	4435	8551	2164

665.238	32	31270	4387	13361	3332
672.102	7	1949	3603	2984	1025
673.252	7	587	4703	3033	692
678.189	9	5981	4466	3644	932
687.234	7	144	4638	2996	731
689.252	149	74928	4562	62844	16045
711.251	7	267	5165	3071	690
721.229	18	8556	4429	7489	2093
724.213	8	1573	4900	3237	824
726.205	7	931	4881	3095	789
776.160	13	2823	4416	5460	1800
804.199	229	35873	5082	94918	27362
806.215	19	7405	3945	7998	3210
826.192	23	6718	4873	9378	3102
842.410	15	344	2173	6228	4143
848.155	8	1374	3649	3352	1678
898.399	7	619	4135	2817	1127
917.461	7	876	4513	2887	1108
922.447	9	2309	5109	3861	1258
927.457	46	65777	5478	19089	6101
1010.450	7	1729	5478	3017	1075
1052.461	7	3137	4957	2867	1309
1109.498	8	2812	4707	3375	1743
1138.507	7	1261	5213	3009	1426
1146.566	8	3183	5442	3217	1480
1163.605	21	15913	4823	8449	4461
1185.625	6	658	6586	2457	938

1222.527	7	2793	5270	2841	1544
1249.630	13	25303	5760	5397	2600
1252.609	9	5591	5194	3582	1986
1269.629	10	3831	5170	4113	2453
1305.720	11	1340	5572	4554	2465
1383.705	8	2225	5399	3245	2036
1399.708	10	12288	5564	3882	2380
1415.701	10	2927	5192	3653	2569
1419.724	20	19469	5551	7621	4893
1425.740	7	4661	4913	2666	2034
1427.743	8	8775	5951	2888	1712
1439.834	44	49935	5692	16794	10832
1447.783	20	14646	5863	7491	4743
1473.760	7	513	6410	2611	1654
1474.892	9	197	13715	3421	1007
1475.799	25	2660	5261	9443	6938
1479.823	254	95627	5697	95734	65360
1501.808	12	11202	6970	4632	2657
1517.786	9	5831	6909	3395	1897
1537.807	11	8049	5520	3980	3190
1567.776	453	385235	5222	165131	140790
1589.763	17	13744	6742	5972	4172
1593.826	8	1572	4694	2767	2922
1597.780	20	28869	4936	7269	7128
1605.731	7	2044	5478	2501	2245
1624.816	16	23187	5603	5638	4746
1639.959	42	73877	5656	14885	12354

1651.826	20	27762	5538	6894	6332
1724.859	10	7412	6291	3251	2746
1880.931	56	165767	6198	16555	16795
1888.946	17	38590	5740	5029	5699
1907.944	47	154748	5690	13328	14971
1910.901	8	5623	5097	2223	2963
1923.947	9	1633	4566	2329	3620
1939.999	6	608	1772	1270	4913
1994.019	24	63007	5982	6196	6923
2019.983	8	5054	4893	1932	2843
2045.066	138	242379	6093	33647	38036
2067.036	9	12451	7476	2050	1958
2083.012	7	9730	6939	1532	1599
2102.063	8	1544	5515	1796	2463
2120.016	12	74753	6263	2603	3010
2150.112	12	48369	5785	2521	3231
2211.108	25	65963	6294	4996	6025
2235.149	12	6481	4847	2211	3778
2283.160	18	11243	5027	3234	5435
2399.069	12	41338	5606	1770	2720
2492.286	18	15435	5323	2388	4293
2509.181	10	1108	4912	1183	2426
2519.333	14	17392	5210	1758	3217
2663.314	9	9752	5598	937	1769
2690.295	8	1934	4951	750	1716
2705.251	17	27882	5631	1800	3345
2748.391	6	2116	5110	598	1152

2757.469	10		10187		5013		981		1968
2807.363	15		6958		4921		1269		3110
2922.456	7	959		4600		461		1302	
3011.581	7	819		5198		480		1252	
3016.679	21		4642		5001		1328		3572
3034.562	9	1732		5779		583		1236	
3122.579	3		7390		736		382		
3455.831	1		8373		199		105		
3477.888	2		7258		263		147		
3487.915	3		7171		465		276		
3511.824	14		13705		5939		520		1420
3753.121	2		6638		251		160		
3809.097	15		2818		5817		361		1193
4945.047	1		18170		54.1		27.4		
5005.300	3		10697		122		90.8		
5056.095	7		4614		318		419		
5110.638	2		8108		84.4		55.9		

Sample 9

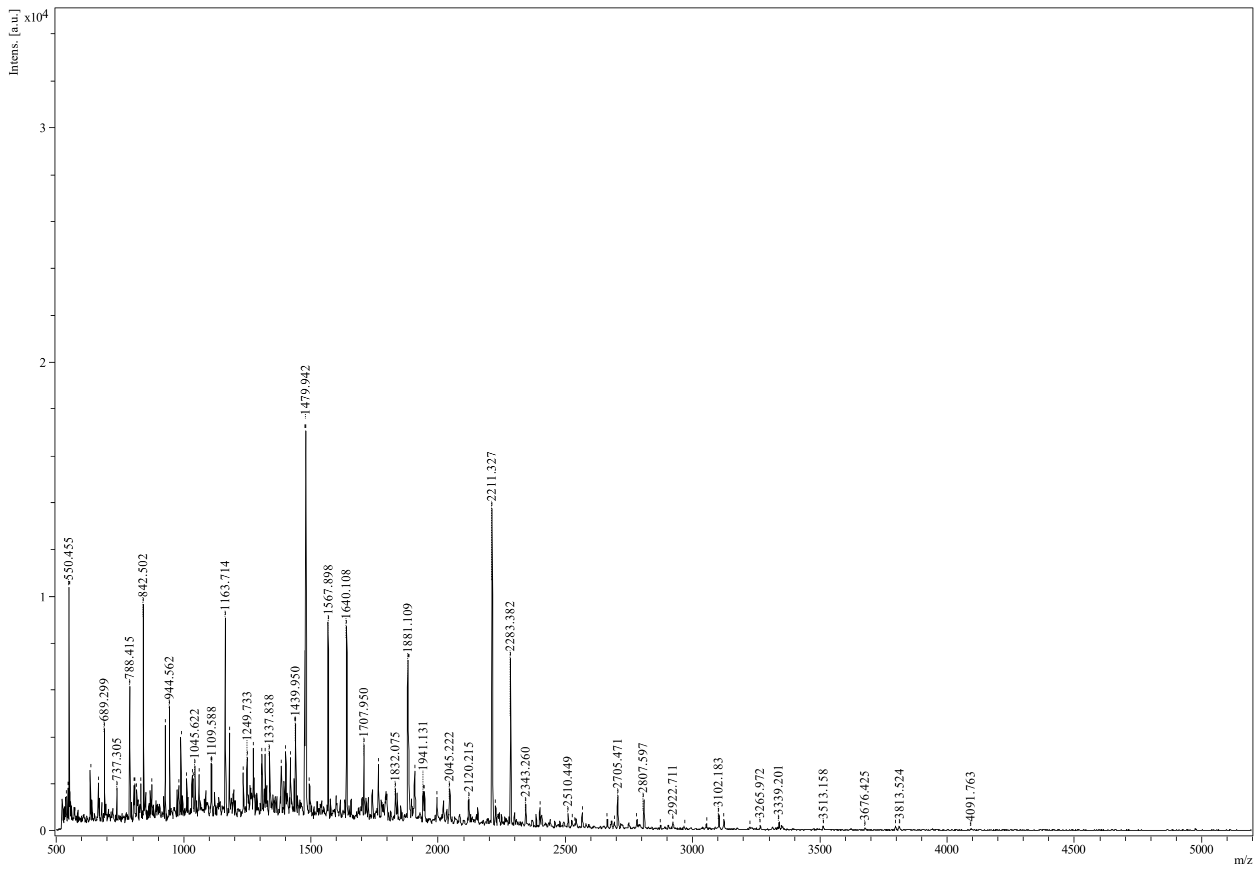

m/z	S/N	Quality Fac.	Res.	Intens.	Area
538.788	6	488	3405	1354	361
544.842	7	322	3061	1506	436
550.455	48	5670	3975	10328	2259
553.222	6	2226	2575	1298	435
634.157	10	3131	3370	2297	787
665.290	8	1694	3902	1702	496
689.299	18	19578	3733	4057	1320
737.305	7	2135	3927	1568	548
788.415	26	16327	3646	6039	2467
804.253	7	1611	2740	1678	1018
807.386	7	1958	3924	1649	644

816.423	6	800		3382		1443		690	
831.454	6	1802		3643		1476		674	
842.502	38		36230		4077		8960		3705
874.448	7	1251		3612		1670		821	
927.527	16		22277		4196		4156		1905
944.562	20		16393		4609		5079		2138
980.559	7	577		4219		1810		898	
989.621	14		16546		4445		3584		1695
1012.514	7	2508		4319		1769		915	
1033.583	7	2973		3755		1876		1165	
1036.580	7	1048		5141		1741		786	
1045.622	8	4107		4534		2132		1108	
1060.617	7	3278		4955		1879		899	
1107.644	6	1449		3769		1672		1174	
1109.588	8	1521		4555		2168		1220	
1163.714	33		15275		5184		8968		4669
1179.702	10		2818		5947		2620		1246
1180.704	9	4837		4782		2355		1407	
1234.768	7	3383		4203		1948		1440	
1249.733	9	2682		5325		2509		1481	
1274.760	9	9268		4328		2476		1853	
1305.824	8	4216		4395		2205		1725	
1307.799	8	4121		4622		2182		1629	
1320.726	9	5577		4496		2523		1960	
1337.838	11		4203		4720		3067		2301
1383.818	9	5974		5003		2375		1808	
1400.823	11		11920		5167		2804		2102

1419.832	9	4384	4913	2470	2009
1434.885	6	3094	5186	1593	1252
1439.950	14	26624	4389	3681	3399
1475.914	27	25956	4698	7039	6256
1479.942	68	78345	5138	17429	14130
1493.885	6	2214	4936	1563	1393
1567.898	34	113929	5464	8279	6968
1640.108	35	129735	5109	8091	8198
1707.950	14	27463	4830	3066	3610
1765.924	11	32854	4478	2219	2983
1832.075	7	6658	5574	1364	1568
1881.109	34	143637	5578	6295	7601
1884.137	11	16651	5174	2039	2688
1907.115	10	1135	4061	1605	2844
1941.131	7	10885	5590	1273	1585
1946.170	7	6525	4968	1181	1673
1994.157	6	1952	4868	908	1370
2045.222	9	7847	4868	1204	1880
2120.215	7	14584	5487	893	1263
2211.327	94	163952	5532	10515	15224
2225.338	7	17316	5453	720	1049
2283.382	47	22693	5224	4762	7910
2285.432	16	645	9861	1692	1282
2343.260	7	5834	4188	674	1377
2399.279	7	6858	4245	599	1201
2510.449	3	6101	633	307	
2525.465	2	6154	433	208	

2537.464	2	5847	295	136	
2565.434	6	12013	4042	422	976
2663.571	2	5737	328	167	
2692.605	2	7087	375	176	
2705.471	16	22019	4847	886	1818
2780.686	3	6702	398	250	
2807.597	16	4212	4747	715	1801
2873.763	2	6433	233	129	
2922.711	3	6397	391	221	
2968.933	1	5305	116	77.5	
3053.979	3	8413	276	171	
3102.183	11	9181	4468	338	1018
3121.879	8	4138	4302	242	781
3223.786	2	5869	122	85.1	
3265.972	3	7656	215	117	
3339.201	4	8341	277	186	
3513.158	3	6896	188	126	
3676.425	1	13249	50.9	18.1	
3796.362	3	7022	148	110	
3813.524	4	8625	153	113	
4091.763	1	8541	40.5	24.1	

Deciphering the mechanoresponsive role of β-catenin in Keratoconus epithelium

Chatterjee Amit [1,2], Prema Padmanabhan[3], Janakiraman Narayanan[#1]

[1] Department of Nanobiotechnology, Vision Research Foundation, Sankara Nethralaya campus, Chennai, Tamil Nadu, India

[2] School of Chemical and Biotechnology, SASTRA, Deemed University, Tanjore, Tamil Nadu, India

[3] Department of Cornea, Medical Research Foundation, Sankara Nethralaya campus, Chennai, Tamil Nadu, India

Running Title:β-catenin mechanotransduction in kerataconus

Correspondence: [#]Dr.Janakiraman Narayanan, Department of Nanobiotechnology, KNBIRVO Block, Vision Research Foundation, Sankara Nethralaya campus, 18/41, College road Nungambakkam, Chennai, Tamil Nadu, India 600006, Tel-+91-44-28271616, (Ext) 1358, Fax-+91-44-28254180, Email: drjrn15@gmail.com

S6

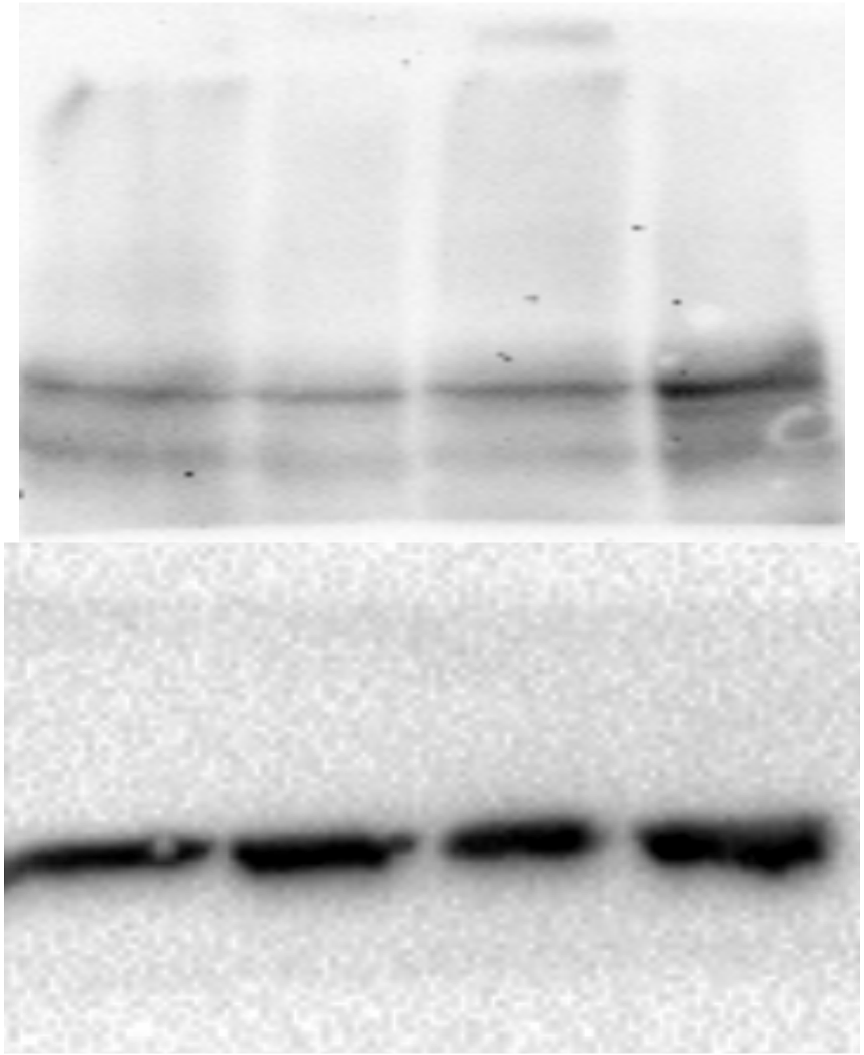

Represented in Fig1B

Represented in Fig1D

Severe Moderate Mild Control

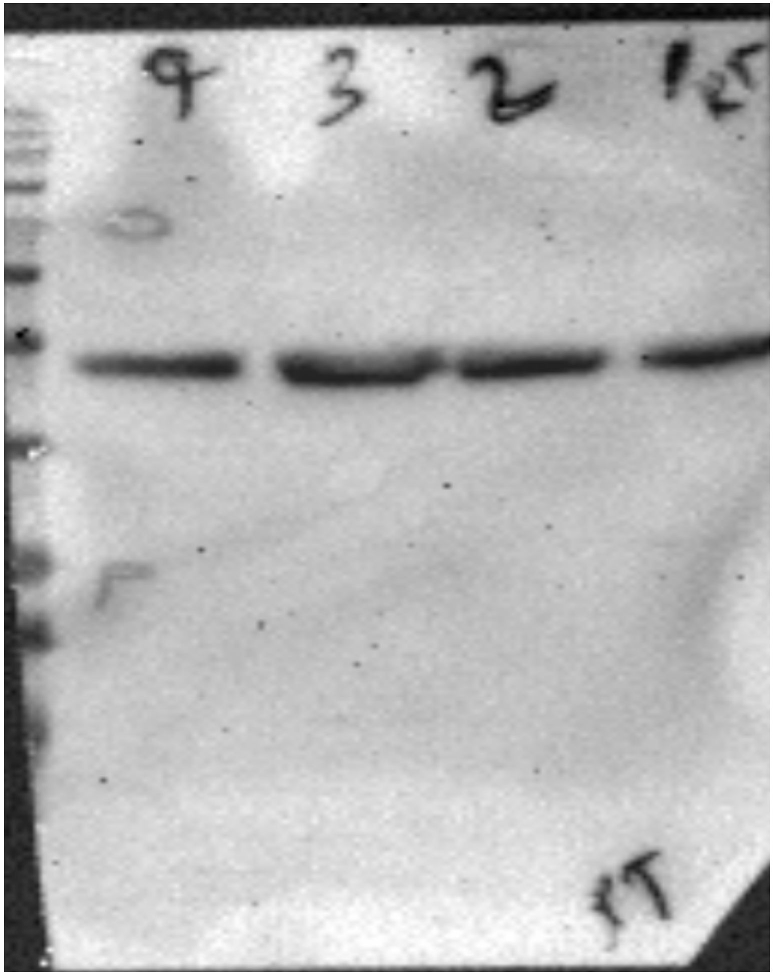

Beta Actin

Severe Moderate Mild Control

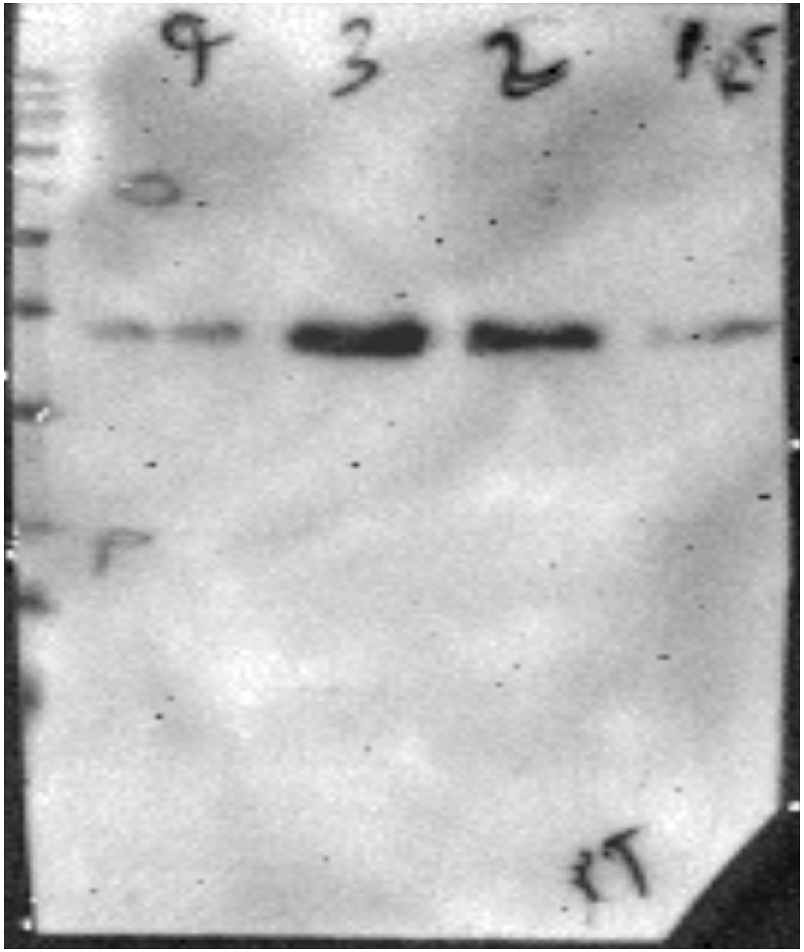

Cyclin D1

S8

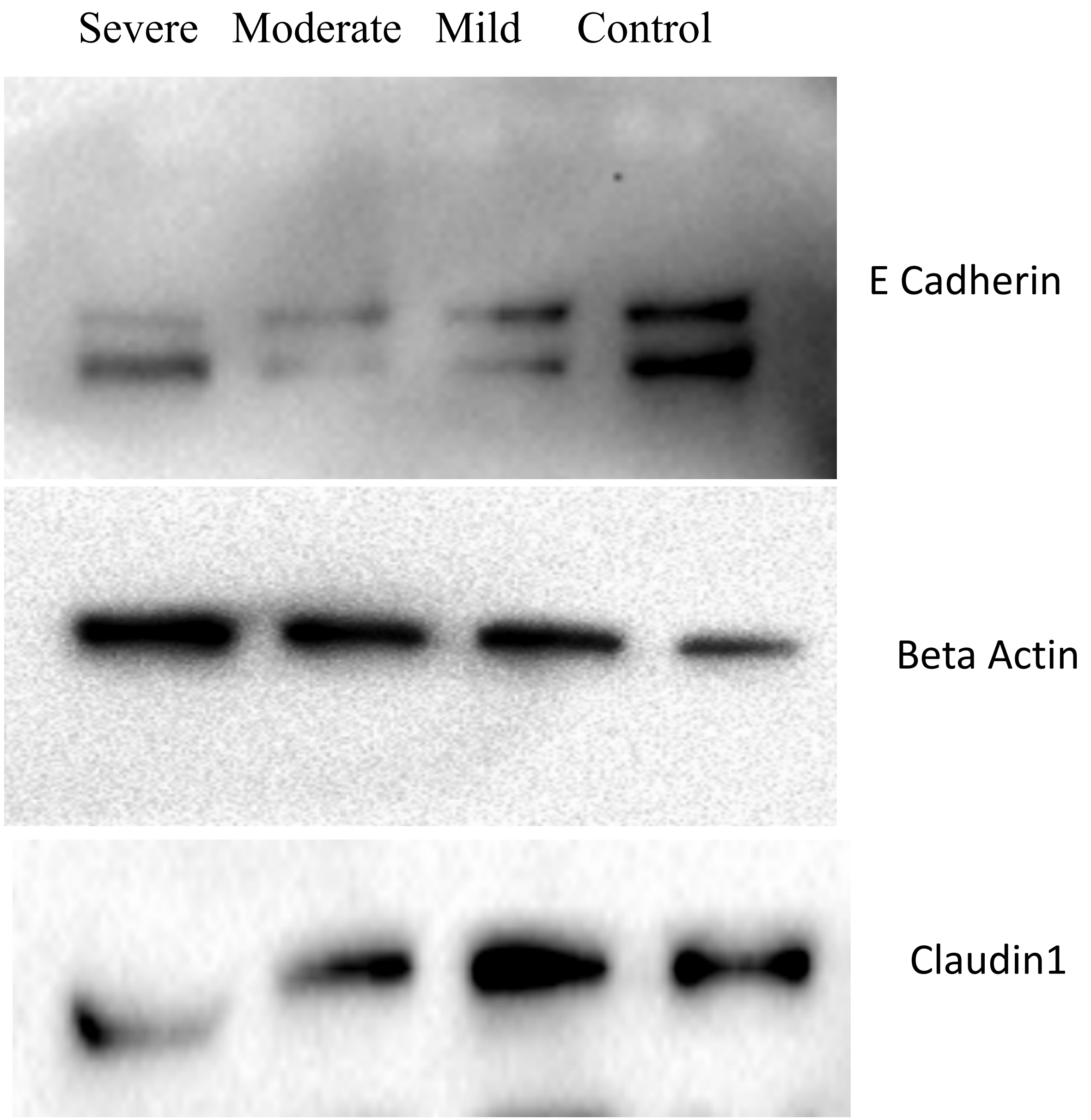

Represented in Fig 2A

S9

Severe Moderate Mild Control

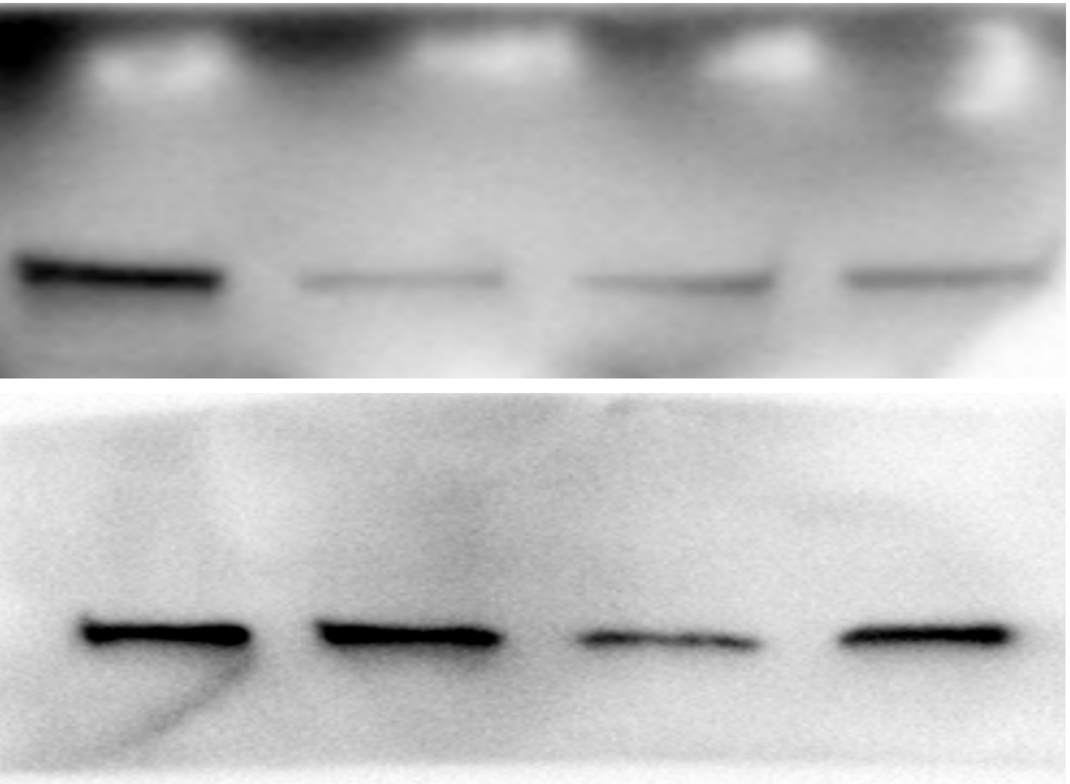

PAN Cadherin

Beta Actin

Represented in Fig 2C

S10

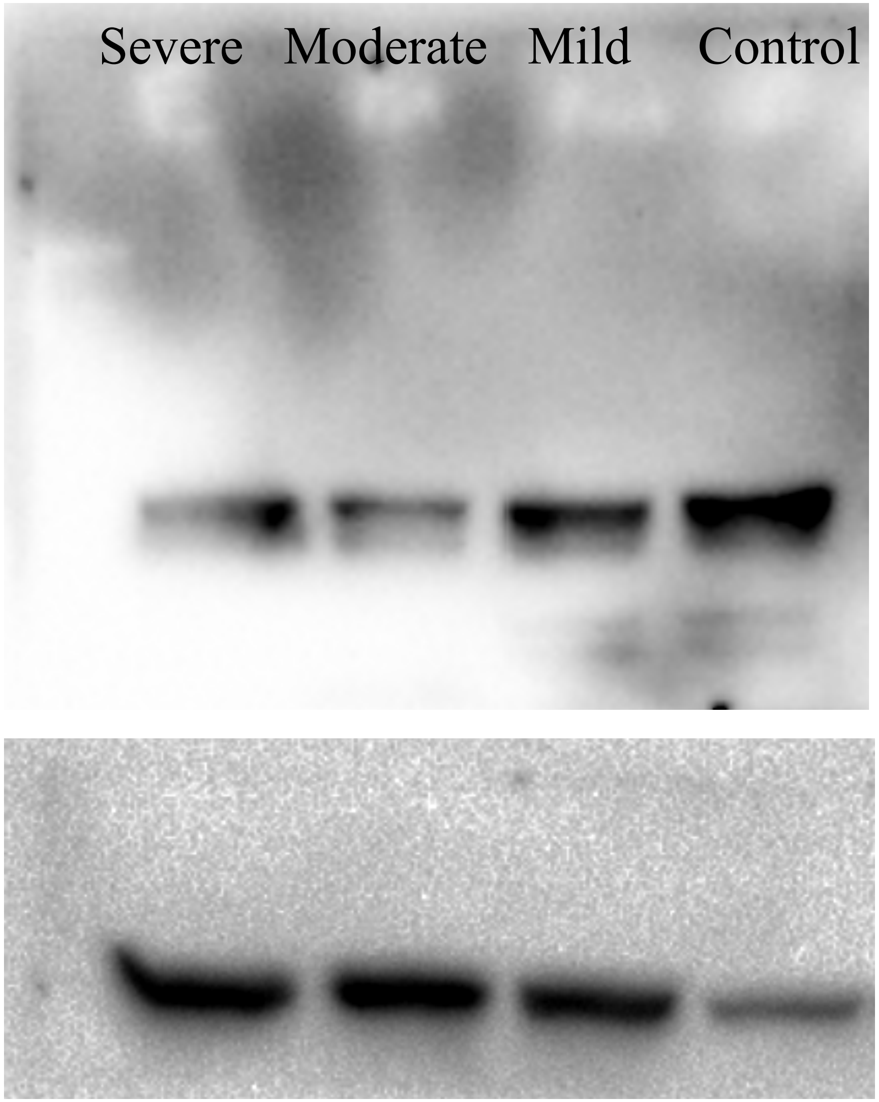

Alpha Catenin

Beta Actin

Represented in Fig 2D

S11

Severe Moderate Mild Control

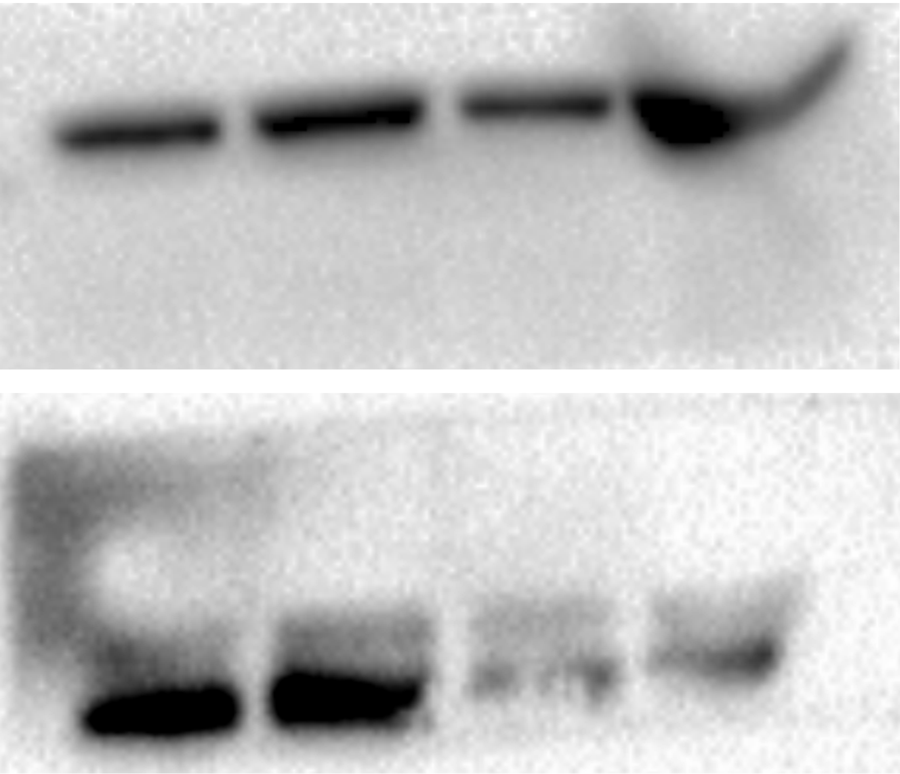

Beta Actin

Rho A

Represented in Fig3B

S12

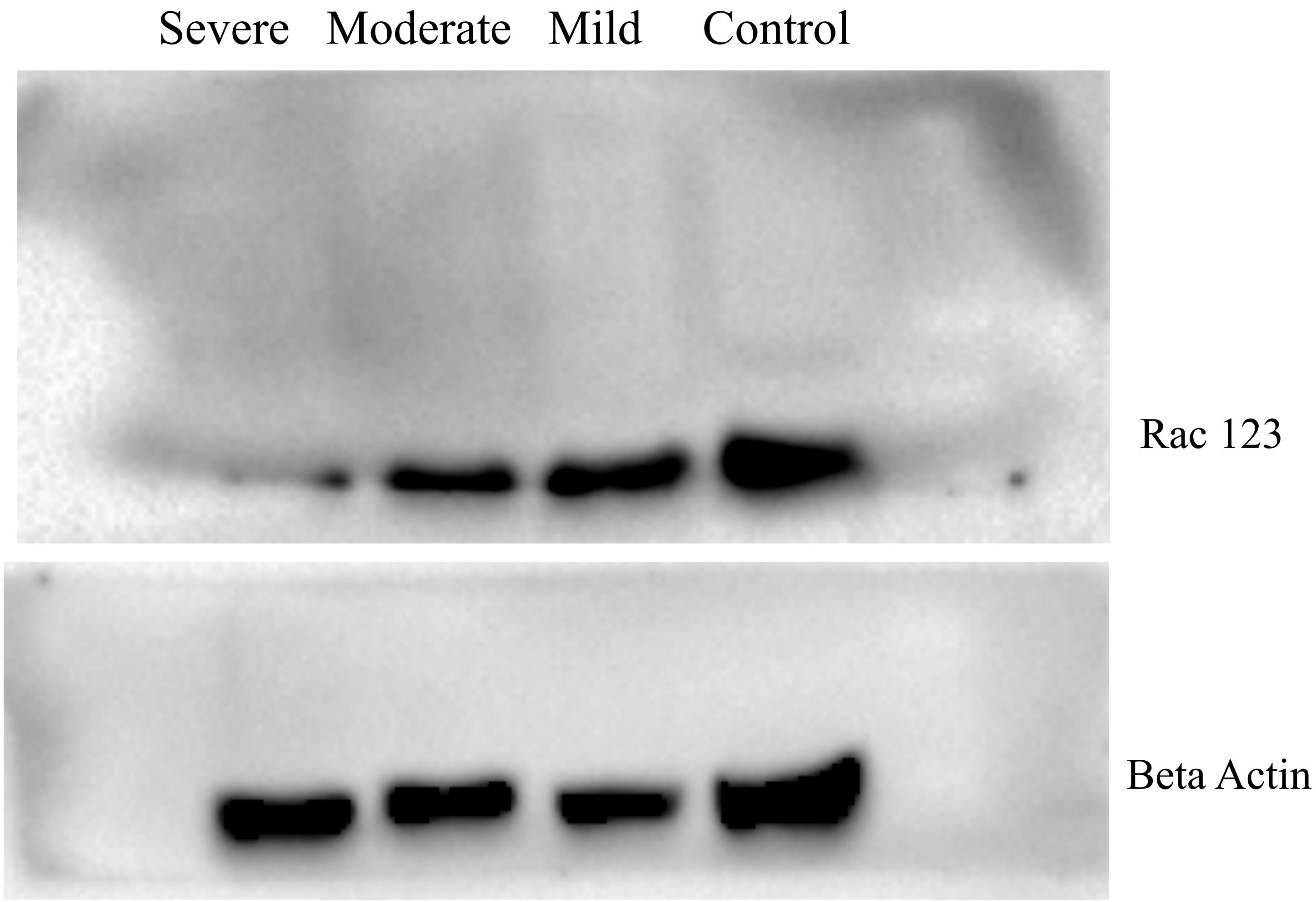

Represented in Fig3C

S13

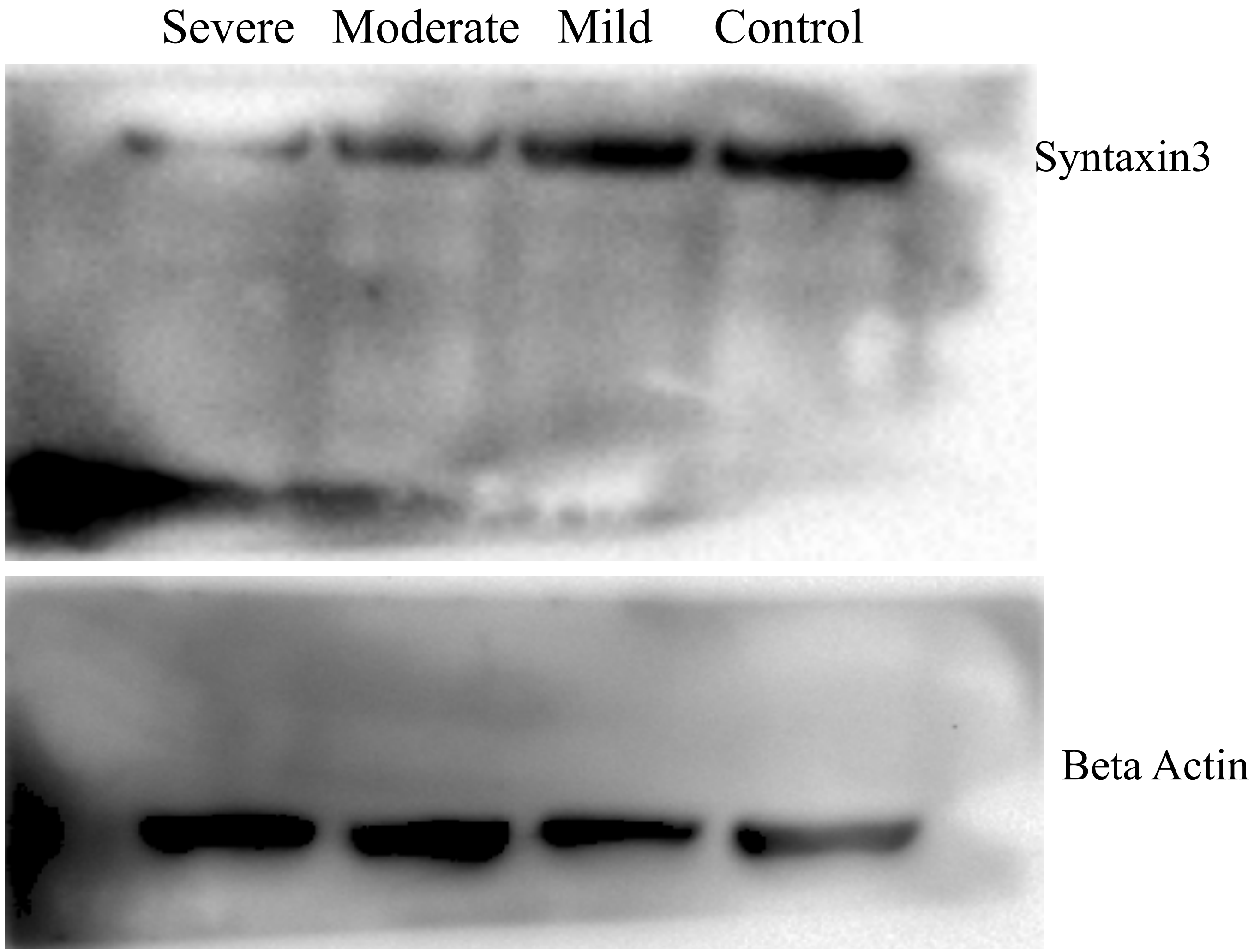

Represented in Fig 3D

S14

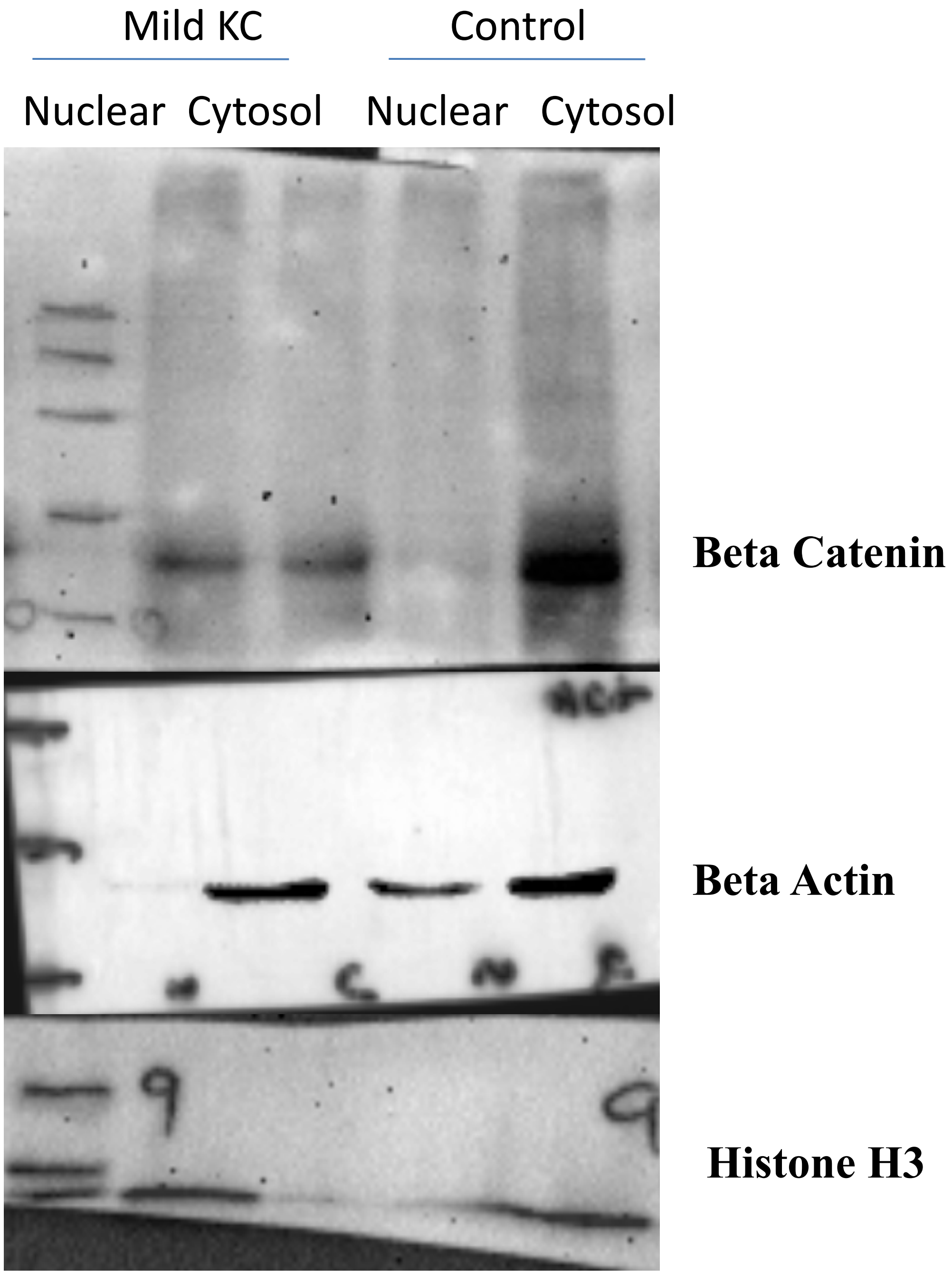

Represented in Fig 4A

S15

Mild Control

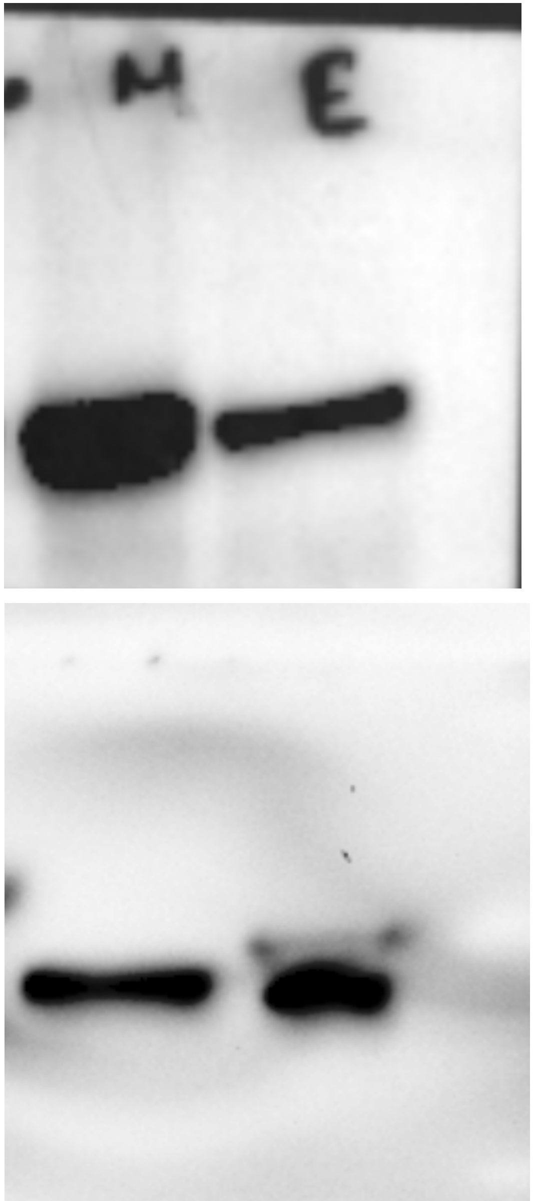

Alpha Actinin

Beta Actin

Represented in Fig4B

S16

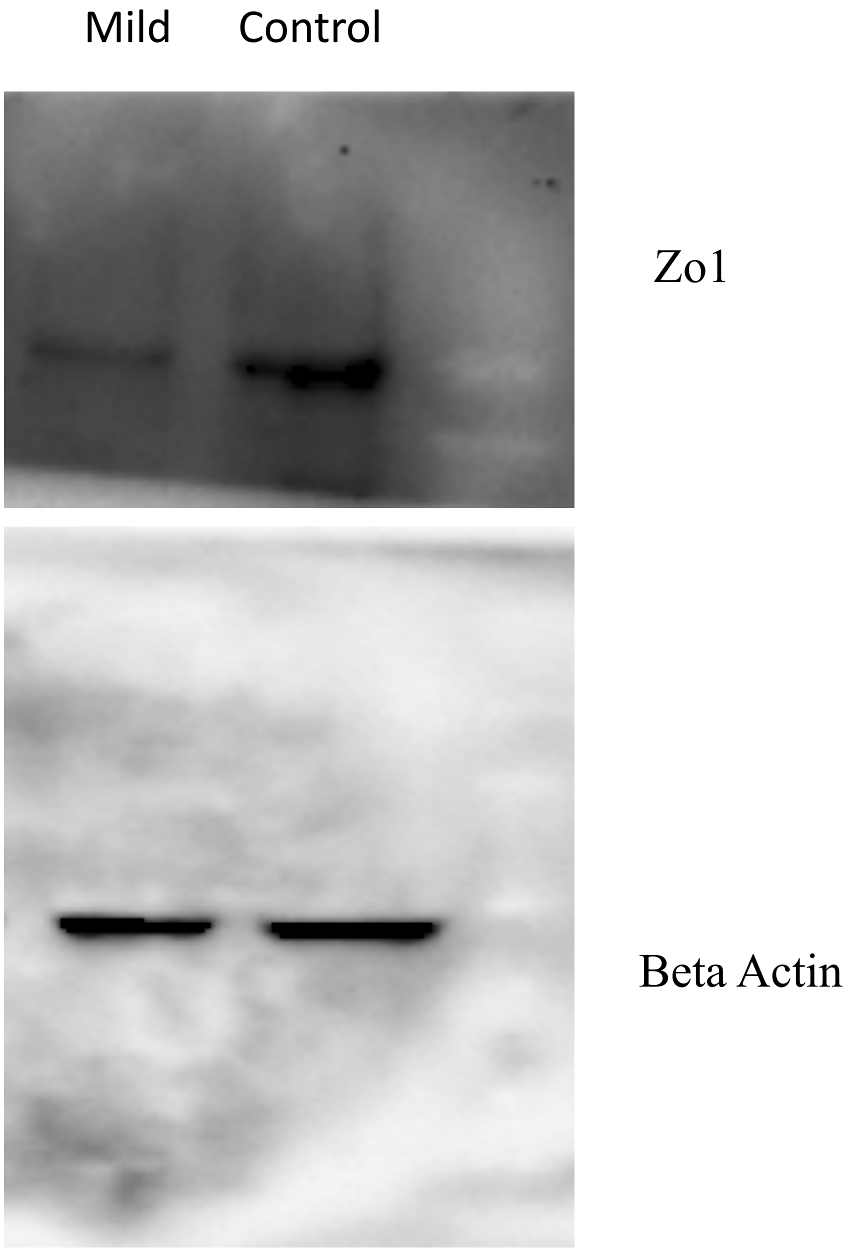

Represented in Fig 4C

Represented in Fig 4D

S17

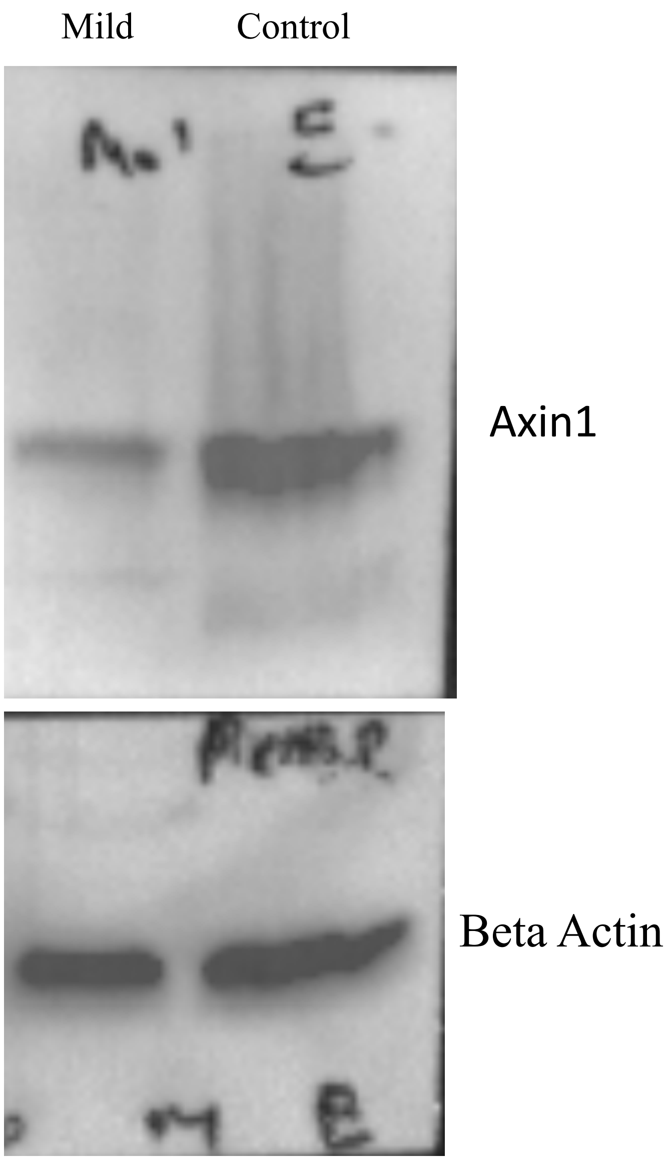

S18

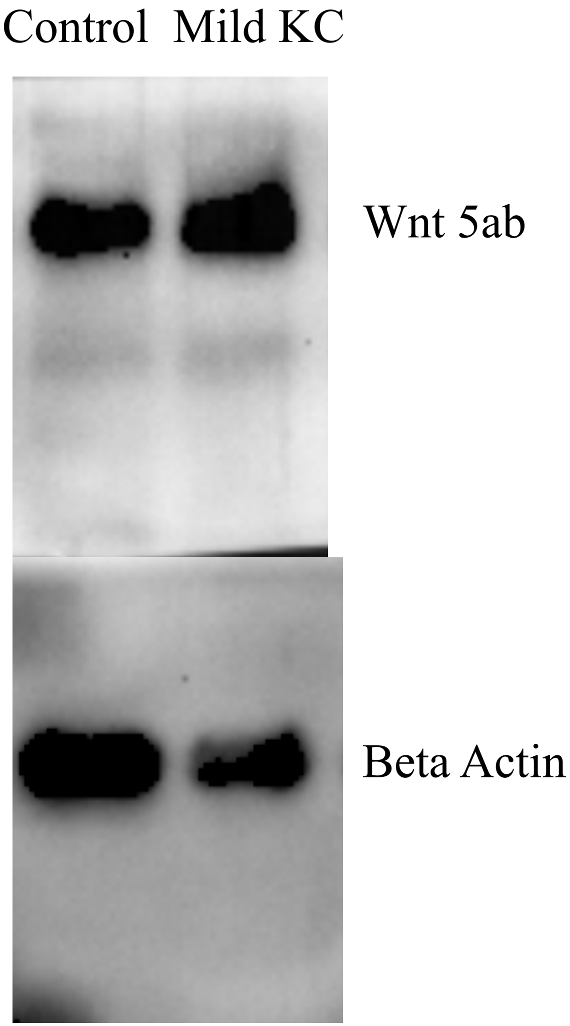

Represented in Fig 5A

Mild IP Control IP IgG Input

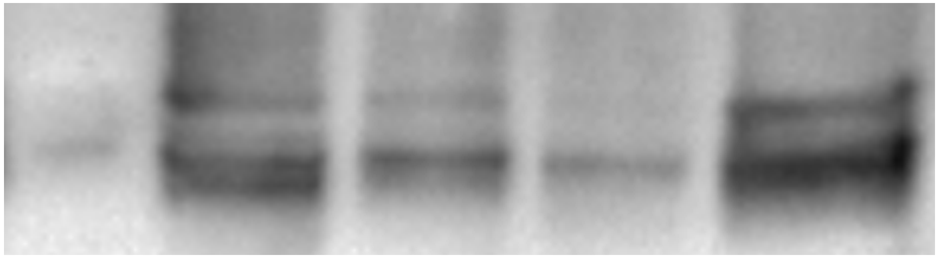

Beta catenin

Represented in Fig 5B

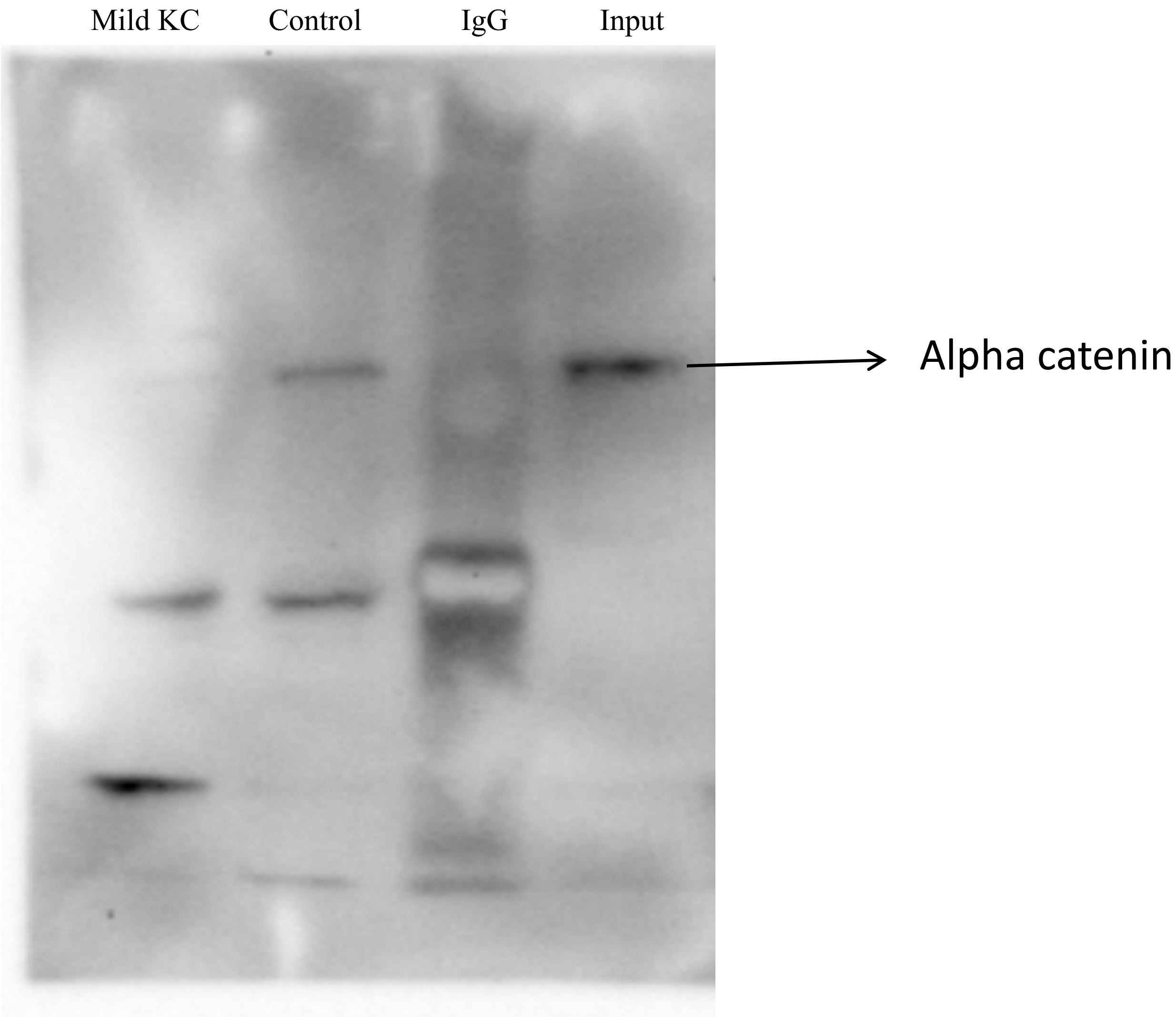

Represented in Fig 5C

S21

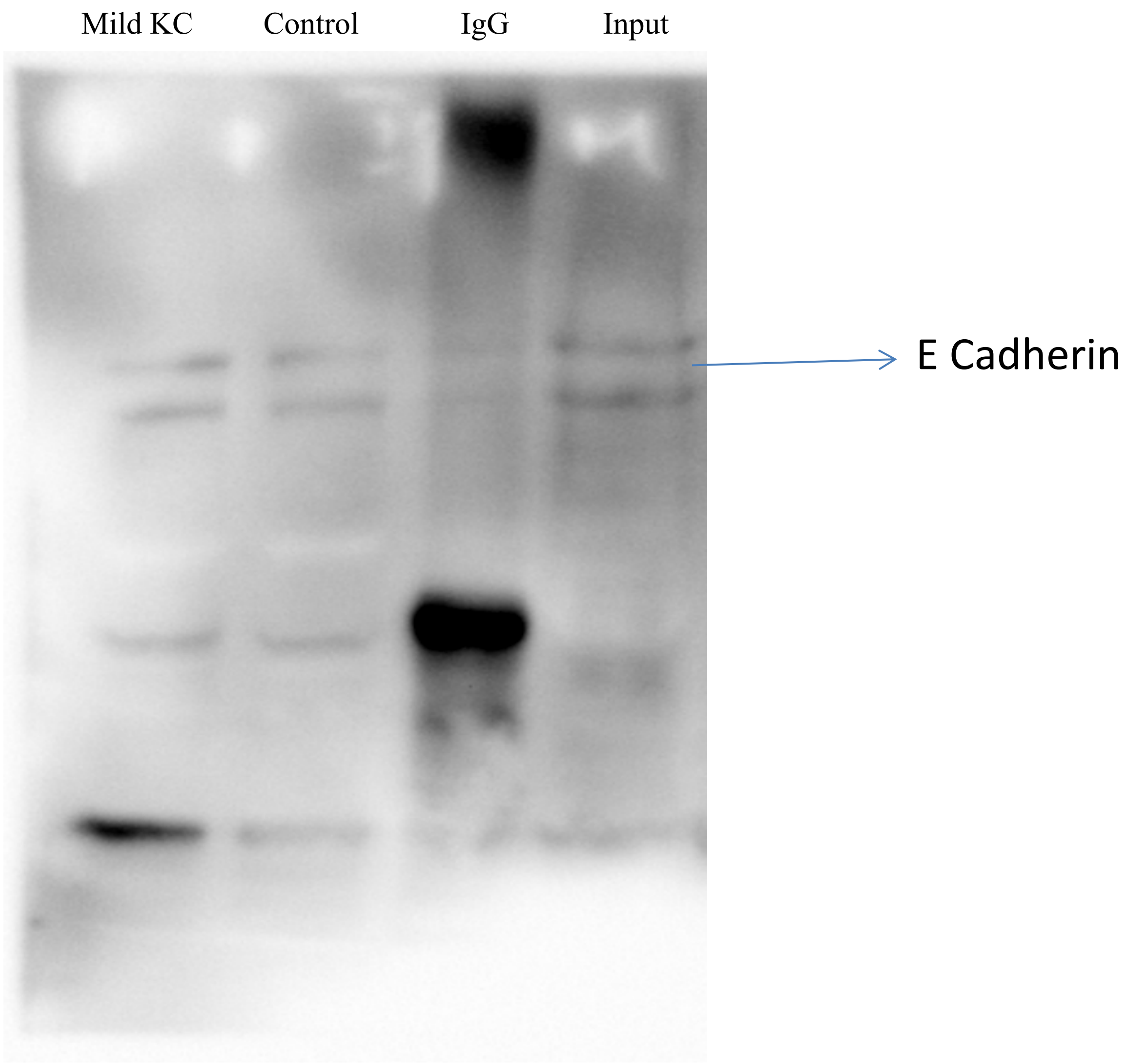

Represented in Fig 5C

S22

Mild Control

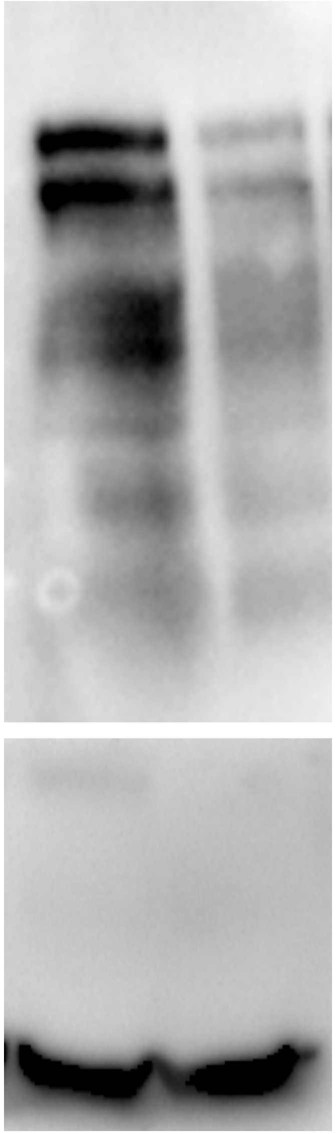

Tenascin C

Beta Actin

Represented in Fig 6A
